# Stabilization of heterochromatin by CLOCK promotes stem cell rejuvenation and cartilage regeneration

**DOI:** 10.1038/s41422-020-0385-7

**Published:** 2020-07-31

**Authors:** Chuqian Liang, Zunpeng Liu, Moshi Song, Wei Li, Zeming Wu, Zehua Wang, Qiaoran Wang, Si Wang, Kaowen Yan, Liang Sun, Tomoaki Hishida, Yanning Cai, Juan Carlos Izpisua Belmonte, Pedro Guillen, Piu Chan, Qi Zhou, Weiqi Zhang, Jing Qu, Guang-Hui Liu

**Affiliations:** 1 State Key Laboratory of Membrane Biology, Institute of Zoology, Chinese Academy of Sciences, Beijing, 100101 China; 2 University of Chinese Academy of Sciences, Beijing, 100049 China; 3 State Key Laboratory of Stem Cell and Reproductive Biology, Institute of Zoology, Chinese Academy of Sciences, Beijing, 100101 China; 4 Institute for Stem Cell and Regeneration, CAS, Beijing, 100101 China; 5 Advanced Innovation Center for Human Brain Protection, National Clinical Research Center for Geriatric Disorders, Xuanwu Hospital Capital Medical University, Beijing, 100053 China; 6 CAS Key Laboratory of Genomic and Precision Medicine, Beijing Institute of Genomics, Chinese Academy of Sciences, Beijing, 100101 China; 7 The MOH Key Laboratory of Geriatrics, Beijing Hospital, National Center of Gerontology, Beijing, 100730 China; 8 Gene Expression Laboratory, Salk Institute for Biological Studies, La Jolla, CA 92037 USA; 9 Clinica Cemtro, Av. del Ventisquero de la Condesa, 42, Madrid, 28035 Spain; 10 China National Center for Bioinformation, Beijing, 100101 China

**Keywords:** Senescence, Mesenchymal stem cells

## Abstract

Accumulating evidence indicates an association between the circadian clock and the aging process. However, it remains elusive whether the deregulation of circadian clock proteins underlies stem cell aging and whether they are targetable for the alleviation of aging-associated syndromes. Here, we identified a transcription factor-independent role of CLOCK, a core component of the molecular circadian clock machinery, in counteracting human mesenchymal stem cell (hMSC) decay. CLOCK expression was decreased during hMSC aging. In addition, CLOCK deficiency accelerated hMSC senescence, whereas the overexpression of CLOCK, even as a transcriptionally inactive form, rejuvenated physiologically and pathologically aged hMSCs. Mechanistic studies revealed that CLOCK formed complexes with nuclear lamina proteins and KAP1, thus maintaining heterochromatin architecture and stabilizing repetitive genomic sequences. Finally, gene therapy with lentiviral vectors encoding CLOCK promoted cartilage regeneration and attenuated age-related articular degeneration in mice. These findings demonstrate a noncanonical role of CLOCK in stabilizing heterochromatin, promoting tissue regeneration, and mitigating aging-associated chronic diseases.

## Introduction

The core circadian machinery is an evolutionarily conserved biological timing system responsible for synchronizing mammalian physiology and behaviors with the external light-dark cycle and stabilizing tissue and cellular homeostasis.^1,2^ Emerging evidence suggests a close link between the circadian clock and the aging process. In mice, age-dependent degradation of the core circadian machinery is observed in the suprachiasmatic nucleus, the major circadian pacemaker, as well as in peripheral tissues.^3–7^ In human, the dysregulation of the circadian clock is evidenced by phase advancements and amplitude reductions in the rhythms of body temperature and cortisol secretion during aging, along with dampened oscillation of core circadian clock genes in the orbitofrontal cortex.^8^ In addition, circadian disorganization has been shown to increase the risks of stem cell aging and aging-associated chronic disorders, including metabolic diseases, neurodegenerative disorders and osteoarthritis.^5,9–11^ The inextricable relationship between aging and the circadian clock has raised two unsolved questions: how aging affects the circadian clock and how the core molecular clock regulates the aging process. Previous studies indicated that aging reprograms the circadian transcriptome in a highly tissue- and cell-type-specific manner.^12,13^ However, the mechanism by which core circadian clock contributes to human aging remains largely unknown.

CLOCK is a core component of the mammalian molecular circadian clock. It contains a bHLH-PAS domain and forms a heterodimer with BMAL1 to act in the positive limb of the circadian transcription/translation feedback loop and initiate clock-controlled gene transcription.^14–16^ Several studies imply interplay between the CLOCK protein and aging. *CLOCK* single nucleotide polymorphisms (SNPs) are associated with obesity, hyperglycemia and type 2 diabetes-associated cardiovascular diseases, Alzheimer’s disease and the quality of aging in a cohort of nonagenarians.^17–21^ Moreover, CLOCK deficiency leads to reduced lifespan and the occurrence of aging-associated pathologies, including cataracts and dermatitis, in mice.^22^ However, the molecular mechanisms and targets by which CLOCK regulates organismal aging remain largely unexplored.

Stem cell aging is a hallmark for organismal aging and the development of aging-related disorders.^23–29^ For example, both physiological aging and premature aging diseases such as Hutchinson-Gilford progeria syndrome (HGPS) are characterized by the functional decay of mesenchymal stem cell (MSC) populations along with the subsequent development of disorders such as osteoarthritis.^28,30–32^ Among the many factors contributing to MSC decay, heterochromatin disorganization has been indicated to be an important driving factor, and restoration of heterochromatin condensation partially reverses the senescence phenotypes of human mesenchymal stem cells (hMSCs).^23,33–36^ Normal heterochromatin organization is essential for preventing aberrant transcription of repetitive sequences and retrotransposition of transposons such as long interspersed nuclear elements 1 (LINE1) in young cells.^33–35,37–43^ Aberrant upregulation of transcription of repetitive sequences including satellite DNA, ribosomal DNA (rDNA) and transposons, along with increased retrotransposition, has been reported to causally activate the senescence-associated secretory phenotype (SASP) and lead to genomic instability during aging.^41,42,44–47^ However, whether chromatin organization and (epi)genomic stability are regulated by the core transcription factors such as CLOCK, remains unclear.

In this study, we found that circadian transcription factor CLOCK formed complexes with nuclear lamina components and heterochromatin-associated proteins to stabilize heterochromatin and maintain hMSCs at a young state independently of its transcriptional activity. Downregulation of CLOCK during hMSC senescence resulted in the destabilization of heterochromatin, transcription of repetitive genomic sequences, and acceleration of cellular senescence. Restored expression of CLOCK or heterochromatin-associated proteins rescued aging defects in aged hMSCs. More importantly, gene therapy with lentiviral vectors encoding CLOCK promoted tissue rejuvenation and alleviated age-related articular degeneration in mice. These results suggest a noncanonical role of CLOCK in maintaining nuclear genomic organization and delaying stem cell senescence, identifying a new therapeutic target for combating age-associated disorders.

## Results

### CLOCK is downregulated in aged hMSCs

**Fig. 1:**
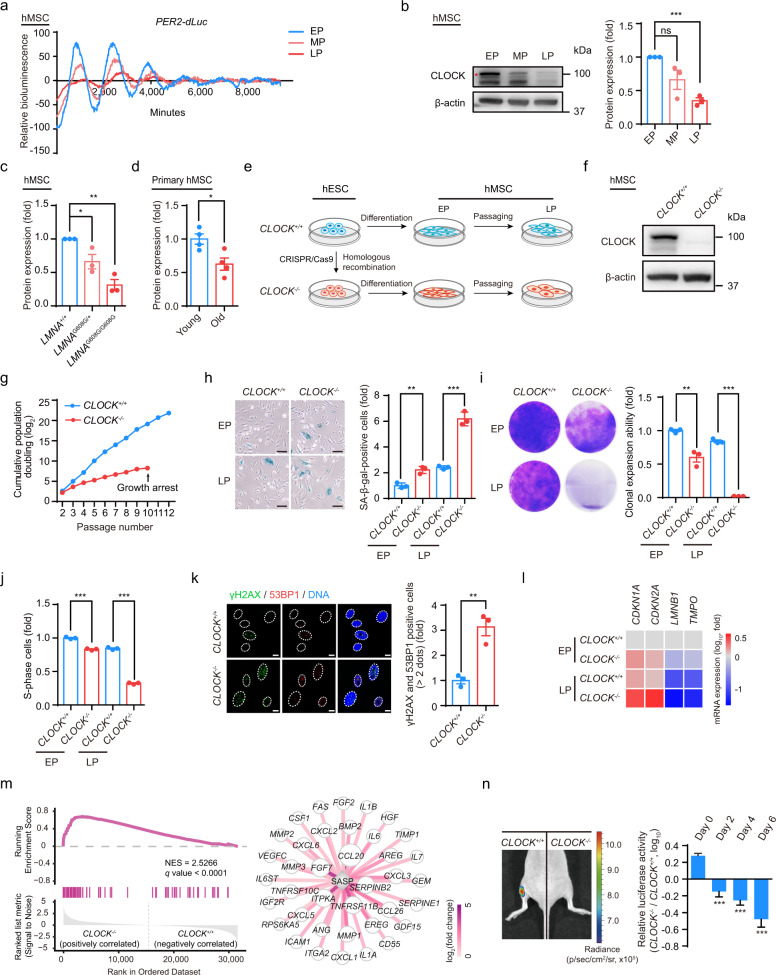
*CLOCK*^−/−^ hMSCs exhibit phenotypes associated with accelerated cellular senescence. **a** Representative traces of circadian oscillation in early-, middle- and late-passage (abbreviated as EP (P4), MP (P9) and LP (P13), respectively) hMSCs monitored by a transiently transfected reporter plasmid expressing destabilized Luc driven by the *PER2* gene promoter (*PER2-dLuc*). Continuous monitoring of Luc activity revealed significant amplitude dampening and period lengthening of *PER2-dLuc* oscillations in MP and LP hMSCs. **b** Western blot analysis of CLOCK in EP (P4), MP (P9) and LP (P13) hMSCs. β-actin was used as the loading control. Data are representative of three independent experiments. Quantitative data to the right are presented as means ± SEM. *n* = 3 independent experiments. ns nonsignificant; ***P < 0.001 (two-tailed unpaired Student’s *t*-test). The red asterisk indicated the CLOCK band. **c** Statistical analysis of the relative protein expression levels of CLOCK in HGPS-specific (*LMNA*^G608G/+^ and *LMNA*^G608G/G608G^) hMSCs. Data are presented as means ± SEM. *n* = 3 independent experiments. *P < 0.05; **P < 0.01 (two-tailed unpaired Student’s *t*-test). **d** Statistical analysis of the relative protein expression levels of CLOCK in young and old primary hMSCs. Data are presented as means ± SEM. *n* = 4 biological replicates. *P < 0.05 (two-tailed unpaired Student’s *t*-test). **e** Schematic illustration of hESC differentiation into hMSCs. **f** Western blot analysis of CLOCK in early-passage *CLOCK*^*+/+*^ and *CLOCK*^−/−^ hMSCs (P4). β-actin was used as the loading control. Data are representative of three independent experiments. **g** Growth curves of *CLOCK*^*+/+*^ and *CLOCK*^*−/−*^ hMSCs. **h** SA-β-gal staining of EP (P4) and LP (P9) *CLOCK*^*+/+*^ and *CLOCK*^*−/−*^ hMSCs. Data are presented as means ± SEM. *n* = 3 biological replicates. **P < 0.01; ***P < 0.001 (two-tailed unpaired Student’s *t*-test). Scale bars, 100 μm. **i** Clonal expansion assay of EP (P4) and LP (P9) *CLOCK*^*+/+*^ and *CLOCK*^*−/−*^ hMSCs. Data are presented as means ± SEM. *n* = 3 biological replicates. **P < 0.01; ***P < 0.001 (two-tailed unpaired Student’s *t*-test). **j** Cell cycle analysis of EP (P4) and LP (P9) *CLOCK*^*+/+*^ and *CLOCK*^*−/−*^ hMSCs. Data are presented as means ± SEM. *n* = 3 biological replicates. ***P < 0.001 (two-tailed unpaired Student’s *t*-test). **k** Immunostaining of γH2AX and 53BP1 in EP (P4) *CLOCK*^*+/+*^ and *CLOCK*^*−/−*^ hMSCs. Dashed lines indicate the nuclear boundaries of the cells. Data are presented as means ± SEM. *n* = 3 biological replicates. **P < 0.01 (two-tailed unpaired Student’s *t*-test). Scale bars, 10 μm. **l** Heatmap showing mRNA levels of the indicated genes in EP (P4) and LP (P9) *CLOCK*^*+/+*^ and *CLOCK*^*−/−*^ hMSCs. The average expression levels of the indicated genes in each cell type were normalized to those in EP *CLOCK*^*+/+*^ hMSCs. Data are representative of two independent experiments. **m** Left, gene set enrichment analysis showing that SASP-associated genes are enriched in late-passage *CLOCK*^*−/−*^ hMSCs compared to *CLOCK*^*+/+*^ hMSCs (P9). Right, network showing the relative expression levels of enriched SASP-associated genes in late-passage *CLOCK*^*−/−*^ hMSCs compared to *CLOCK*^*+/+*^ hMSCs (P9). **n** Photon flux from the TA muscles of NOD-SCID mice transplanted with *CLOCK*^*+/+*^ (left) or *CLOCK*^*−/−*^ hMSCs (right) expressing luciferase. To indicate hMSCs attrition after implantation, Luc activity in the TA muscles was detected with an in vivo imaging system (IVIS). Quantitative data to the right are presented as means ± SEM. *n* = 6 mice. ***P < 0.001 (two-tailed unpaired Student’s *t*-test).

To investigate the changes in the circadian clock during hMSC senescence, we first utilized the early-, middle- and late-passage hMSCs that stably expressed luciferase driven by a *PER2* promoter (*PER2-dLuc*) to measure circadian rhythms affected by replicative senescence. Continuous monitoring of luciferase (Luc) activity revealed amplitude dampening and period lengthening of *PER2-dLuc* reporter oscillations in the middle- and late-passage hMSCs (Fig. 1a). Western blotting revealed that the core circadian transcription factor CLOCK was downregulated in replicative senescent hMSCs, consistent with the decreased amplitude of *PER2-dLuc* oscillations (Fig. 1b). Similarly, CLOCK protein levels were decreased in *LMNA* (p.G608G)-mutant prematurely aging hMSCs (HGPS-specific hMSCs) (Fig. 1c; Supplementary information, Fig. S1g).^28,30^ We also observed a decrease in CLOCK protein levels with age in primary hMSCs derived from healthy human donors of different ages (Fig. 1d; Supplementary information, Fig. S1f). Senescence phenotypes of late-passage hMSCs, HGPS-specific hMSCs, and primary hMSCs derived from elderly individuals were characterized by increased numbers of senescence-associated (SA)-β-gal-positive cells and decreased cellular clonal expansion ability (Supplementary information, Fig. S1a–e). Collectively, these observations indicate that CLOCK may be involved in the regulation of hMSC senescence.

### CLOCK plays a geroprotective role in hMSCs

To study the role of CLOCK in regulating cellular senescence, we first generated CLOCK-deficient human embryonic stem cells (hESCs) by utilizing CRISPR/Cas9-facilitated homologous recombination (HR) to delete the exon 5 of the *CLOCK* gene in hESCs (Supplementary information, Fig. S1h).^48^ Successful gene targeting at the *CLOCK* locus was verified by western blotting (Supplementary information, Fig. S1i). Karyotyping showed that genomic integrity was maintained in *CLOCK*^−/−^ hESCs (Supplementary information, Fig. S1j). Furthermore, CLOCK deficiency did not alter hESC-associated characteristics, such as hESC morphology, DNA hypomethylation at the *OCT4* promoter, pluripotency, or the in vivo differentiation potential towards the three germ layer lineages (Supplementary information, Fig. S1k, n and p). In addition, no differences in cellular proliferation ability or cell cycle kinetics were observed between *CLOCK*^−/−^ and *CLOCK*^+/+^ hESCs (Supplementary information, Fig. S1l, m and o). These results indicate that CLOCK is dispensable for the maintenance of hESC homeostasis.

We next differentiated *CLOCK*^−/−^ hESCs into hMSCs (referred to as *CLOCK*^–/–^ hMSCs), a type of adult stem cells susceptible to senescence (Fig. 1e).^23,49–51^ *CLOCK*^–/–^ hMSCs were positive for typical mesenchymal progenitor markers such as CD73, CD90, CD105, CD44, CD166, CD29, CD13, and HLA-ABC, and negative for non-hMSC markers including CD45, CD34, CD43, CD164, CD14, CD19 and PDPN (Supplementary information, Fig. S2a).^52^ The lack of CLOCK protein in *CLOCK*^−/−^ hMSCs was verified by western blotting (Fig. 1f). Both *CLOCK*^+/+^ and *CLOCK*^–/–^ hMSCs were able to differentiate into osteoblasts, chondrocytes, and adipocytes, though *CLOCK*^–/–^ hMSCs exhibited a mild defect in chondrogenesis (Supplementary information, Fig. S2b). As expected, *PER2-dLuc* reporter assays revealed compromised circadian activation of *PER2* gene transcription in *CLOCK*^–/–^ hMSCs (Supplementary information, Fig. S2c). Monitoring of the transcript levels of key circadian proteins every 3 h after synchronization (JTK_Cycle analysis) revealed that the oscillations of *PER3*, *NR1D1* and *DBP* and the amplitude of *NR1D2* oscillation were compromised in *CLOCK*^–/–^ hMSCs (Supplementary information, Fig. S2d), recapitulating the phenotypes of senescent hMSCs (Supplementary information, Fig. S2e). These cellular circadian defects were rescued by reintroduction of CLOCK into *CLOCK*^−/−^ hMSCs (Supplementary information, Fig. S2d).

To investigate whether CLOCK deficiency mediated hMSC senescence, we subjected *CLOCK*^+/+^ and *CLOCK*^–/–^ hMSCs to serial passaging. Compared with *CLOCK*^+/+^ hMSCs, which exhibited normal aging kinetics, *CLOCK*^–/–^ hMSCs senesced as early as at passage 4 and underwent complete growth arrest at passage 10 (Fig. 1g). Increased SA-β-gal activity, decreased proliferative potential, and decreased percentage of S-phase cells were detected in *CLOCK*^–/–^ hMSCs (Fig. 1h–j). In addition, CLOCK deficiency resulted in the enhancement of DNA damage response and SASP (Fig. 1k, m; Supplementary information, Fig. S2f). Consistent with the accelerated development of senescence phenotypes, we observed downregulated transcription levels of *LMNB1* and *TMPO* and upregulated mRNA expression of the aging markers *CDKN1A* (P21) and *CDKN2A* (P16) in *CLOCK*^–/–^ hMSCs (Fig. 1l). Finally, compared to *CLOCK*^+/+^ cells, *CLOCK*^–/–^ hMSCs exhibited accelerated decay in vivo when implanted into the tibialis anterior (TA) muscles of immunodeficient mice (Fig. 1n).

To further evaluate the direct role of CLOCK in regulating cellular senescence, *CLOCK*^+/+^ hMSCs were transduced with lentiviruses encoding CRISPR/Cas9 and an sgRNA targeting *CLOCK* or a nontargeting control (NTC) sgRNA (Supplementary information, Fig. S2g).^53^ Similar to *CLOCK*^–/–^ hESC-derived hMSCs, hMSCs with CLOCK inactivation exhibited accelerated senescence characterized by increased SA-β-gal activity and decreased clonal expansion ability (Supplementary information, Fig. S2h, i). Taken together, these data indicate that CLOCK is a geroprotective factor in hMSCs.

### Stabilization of heterochromatin by CLOCK counteracts hMSC senescence

**Fig. 2:**
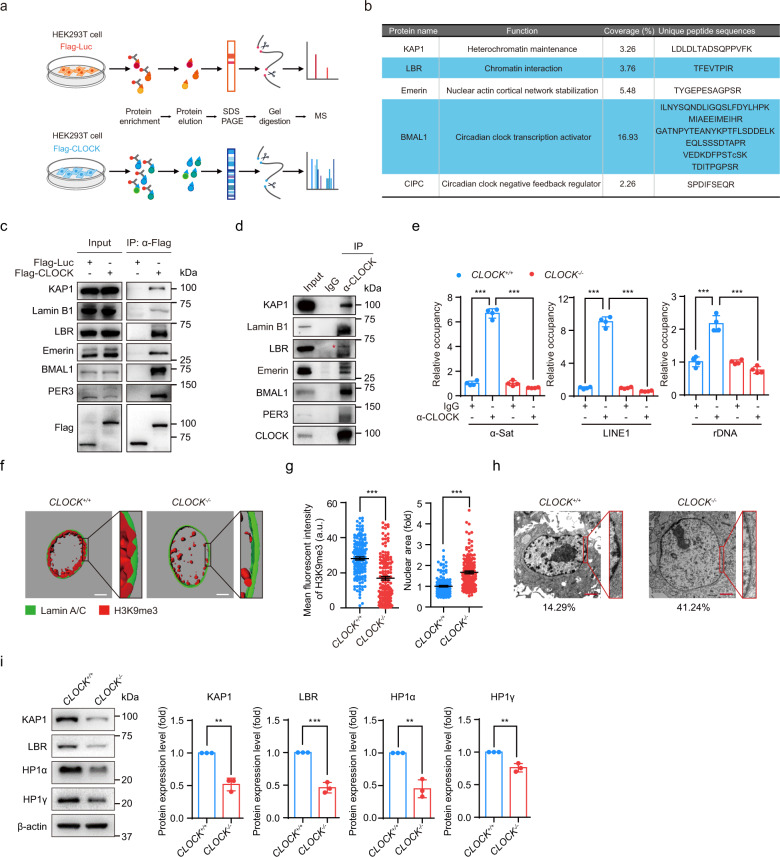
CLOCK forms complexes with nuclear lamina proteins and the heterochromatin-associated protein KAP1. **a** The flow chart of the mass spectrometry strategy for identifying interacting proteins of CLOCK. Luc was used as a control. **b** KAP1, LBR, Emerin, BMAL1 and CIPC were interacting proteins of CLOCK identified by mass spectrometry. Unique peptides of each interacting protein are listed in the table. **c** Co-IP analysis of KAP1, Lamin B1, LBR, Emerin, BMAL1 and PER3 with exogenous Flag-tagged CLOCK protein in HEK293T cells. Data are representative of three independent experiments. **d** Co-IP analysis of KAP1, Lamin B1, LBR, Emerin, BMAL1 and PER3 with endogenous CLOCK protein in *CLOCK*^+/+^ hMSCs. The red asterisk indicates the LBR band pulled down by IP with CLOCK antibody. Data are representative of two independent experiments. **e** Enrichment of CLOCK within α-Sat, LINE1, and rDNA regions, as measured by ChIP-qPCR. Data are presented as means ± SD. *n* = 4. ***P < 0.001 (two-tailed unpaired Student’s *t-*test). Data are representative of two independent experiments. **f** 3D construction of a z-stack of H3K9me3 (red) and Lamin A/C (green) immunofluorescence images of late-passage *CLOCK*^*+/+*^ and *CLOCK*^*−/−*^ hMSCs (P9). Scale bars, 5 μm. **g** Left, mean fluorescence intensity of H3K9me3 in late-passage *CLOCK*^*+/+*^ and *CLOCK*^*−/−*^ hMSCs (P9). Data are presented as means ± SEM. *n* = 150 cells from three biological replicates. ***P < 0.001 (two-tailed unpaired Student’s *t*-test). Right, quantitative nuclear area of late-passage *CLOCK*^*+/+*^ and *CLOCK*^*−/−*^ hMSCs (P9). Data are presented as means ± SEM. *n* = 150 cells from three biological replicates. ***P < 0.001 (two-tailed unpaired Student’s *t*-test). **h** Electron microscopy analysis of the heterochromatin architecture at the nuclear periphery in late-passage *CLOCK*^*+/+*^ and *CLOCK*^*−/−*^ hMSCs (P9). The percentages of cells with heterochromatin loss at the nuclear periphery are presented below the images. Scale bars, 2 μm. **i** Representative western blots of nuclear lamina and heterochromatin components in early-passage *CLOCK*^*+/+*^ and *CLOCK*^*−/−*^ hMSCs (P4). β-actin was used as the loading control. Quantitative data to the right are presented as means ± SEM. *n* = 3 independent experiments. **P < 0.01; ***P < 0.001 (two-tailed unpaired Student’s *t*-test).

To elucidate the molecular mechanism by which CLOCK deficiency accelerated hMSC aging, we sought to identify new CLOCK-interacting proteins by expressing Flag-tagged CLOCK proteins in HEK293T cells and performed immunoprecipitation with an anti-Flag antibody followed by mass spectrometry analysis (IP-MS) (Fig. 2a). Flag-luciferase was used as a negative control. We identified hundreds of proteins, including the well-known circadian clock proteins BMAL1 and CIPC, as CLOCK interaction partners (Fig. 2b; Supplementary information, Table S3). Interestingly, a panel of nuclear lamina proteins, such as LBR and Emerin, as well as the heterochromatin-associated protein KAP1, were also identified in CLOCK protein complexes (Fig. 2b; Supplementary information, Table S3). Co-immunoprecipitation (Co-IP) assays verified these new interactions between CLOCK and nuclear lamina/heterochromatin-associated proteins in HEK293T cells and hMSCs (Fig. 2c, d). We next mapped the domains of CLOCK required for such interactions. To this end, a series of Flag-tagged CLOCK truncation mutants were generated, including amino acids (a.a.) 1–379, a.a. 1–651, a.a. 380–651, a.a. 380–846 and a.a. 652–846 (Supplementary information, Fig. S3a and Table S1). Heterochromatin-associated protein KAP1 and nuclear lamina proteins Lamin B1, LBR, and Emerin interacted with CLOCK via a.a. 1–651. The interactions with KAP1, Lamin B1 and Emerin relied mainly on a.a. 1–379 of CLOCK, and the interaction with LBR relied primarily on a.a. 380–651 of CLOCK (Supplementary information, Fig. S3b, c). As a positive control, only the full-length CLOCK interacted with PER3, another core circadian clock factor regulated by CLOCK (Supplementary information, Fig. S3b).^54,55^ These new interactions identified between CLOCK and heterochromatin components suggest a role of CLOCK in regulating chromatin organization. As KAP1 and Lamin B1 are usually clustered in the repetitive sequences enriched with H3K9me3,^56^ we next sought to explore whether CLOCK was tethered to heterochromatin region by performing chromatin immunoprecipitation-quantitative polymerase chain reaction (ChIP-qPCR). We found that CLOCK bound to heterochromatin-enriched elements such as pericentromeric repetitive sequences (e.g., α-satellite (α-Sat)), long interspersed repetitive elements (e.g., LINE1) and rDNA (Fig. 2e).

**Fig. 3:**
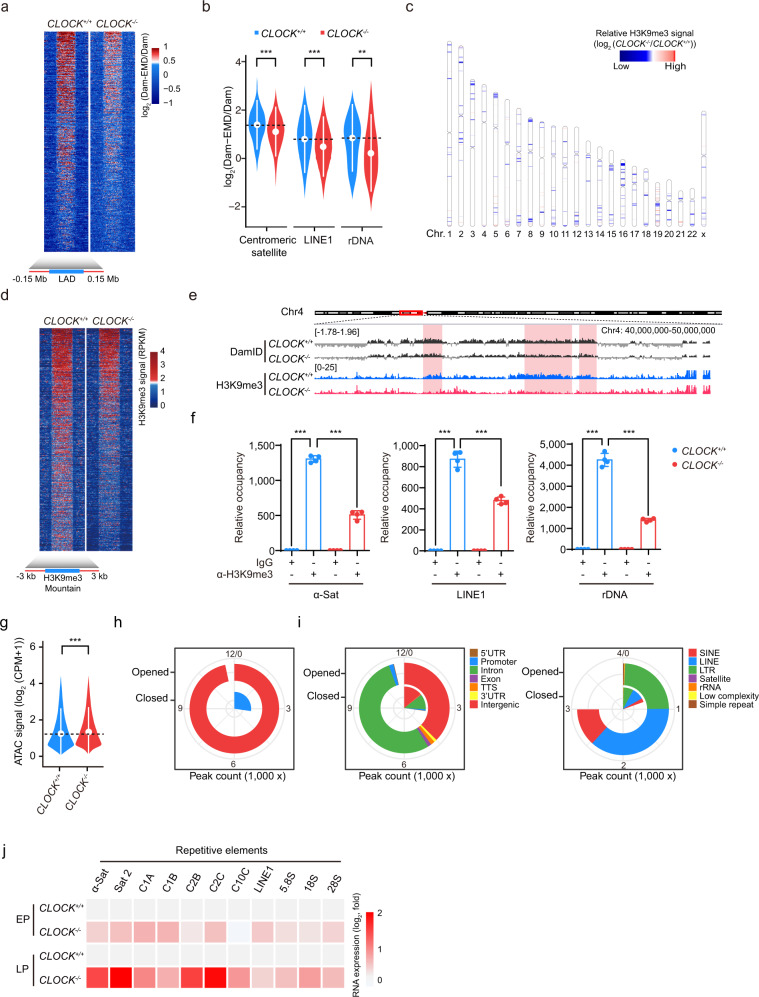
CLOCK is required for heterochromatin maintenance in hMSCs. **a** Heatmap showing the DamID signal (log_2_ (Dam-EMD/Dam)) from 0.15 Mb upstream to 0.15 Mb downstream of LAD regions in late-passage *CLOCK*^*+/+*^ and *CLOCK*^*−/−*^ hMSCs (P9). **b** Violin plot showing the DamID signal (log_2_ (Dam-EMD/Dam)) at the indicated repetitive elements in late-passage *CLOCK*^*+/+*^ and *CLOCK*^*−/−*^ hMSCs (P9). The white circles represent the median values, and the white lines represent the values within the interquartile range (IQR) from smallest to largest. **P < 0.01; ***P < 0.001 (two-sided Wilcoxon rank-sum test). **c** Chromosome ideogram showing the levels of H3K9me3 mountains across the 23 chromosomes of late-passage *CLOCK*^*−/−*^ hMSCs compared to those of *CLOCK*^*+/+*^ hMSCs (P9). The color key from blue to red indicates low to high relative H3K9me3 levels, respectively. **d** Heatmap showing the H3K9me3 signal (RPKM) from 3 kb upstream to 3 kb downstream of H3K9me3 mountain regions in late-passage *CLOCK*^*+/+*^ and *CLOCK*^*−/−*^ hMSCs (P9). **e** Representative tracks of DamID and H3K9me3 ChIP-seq data showing pronounced loss of H3K9me3 signal (RPKM) accompanied by decreased DamID signal (log_2_ (Dam-EMD/Dam)) in *CLOCK*^*−/−*^ hMSCs. **f** Enrichment of H3K9me3 within α-Sat, LINE1, and rDNA regions, as measured by ChIP-qPCR. Data are presented as means ± SD. *n* = 4. ***P < 0.001 (two-tailed unpaired Student’s *t-*test). Data are representative of two independent experiments. **g** Violin plot showing the ATAC signal at the genomic regions of ATAC-seq peaks in late-passage *CLOCK*^*+/+*^ and *CLOCK*^*−/−*^ hMSCs (P9). The white circles represent the median values, and the white lines represent the values within the IQR from smallest to largest. ***P < 0.001 (two-sided Wilcoxon rank-sum test). **h** Pie chart showing the counts of opened and closed ATAC peaks in LP (P9) *CLOCK*^*−/−*^ hMSCs compared to *CLOCK*^*+/+*^ hMSCs. **i** Genomic element enrichment analysis of opened and closed ATAC peaks in LP (P9) *CLOCK*^*−/−*^ hMSCs compared to *CLOCK*^*+/+*^ hMSCs. **j** Heatmap showing the relative mRNA levels of repetitive elements in EP (P4) or LP (P9) *CLOCK*^*+/+*^ and *CLOCK*^−/−^ hMSCs. The average expression levels of the indicated repetitive elements in each cell type were normalized to those in *CLOCK*^*+/+*^ hMSCs. Data are representative of three independent experiments.

**Fig. 4:**
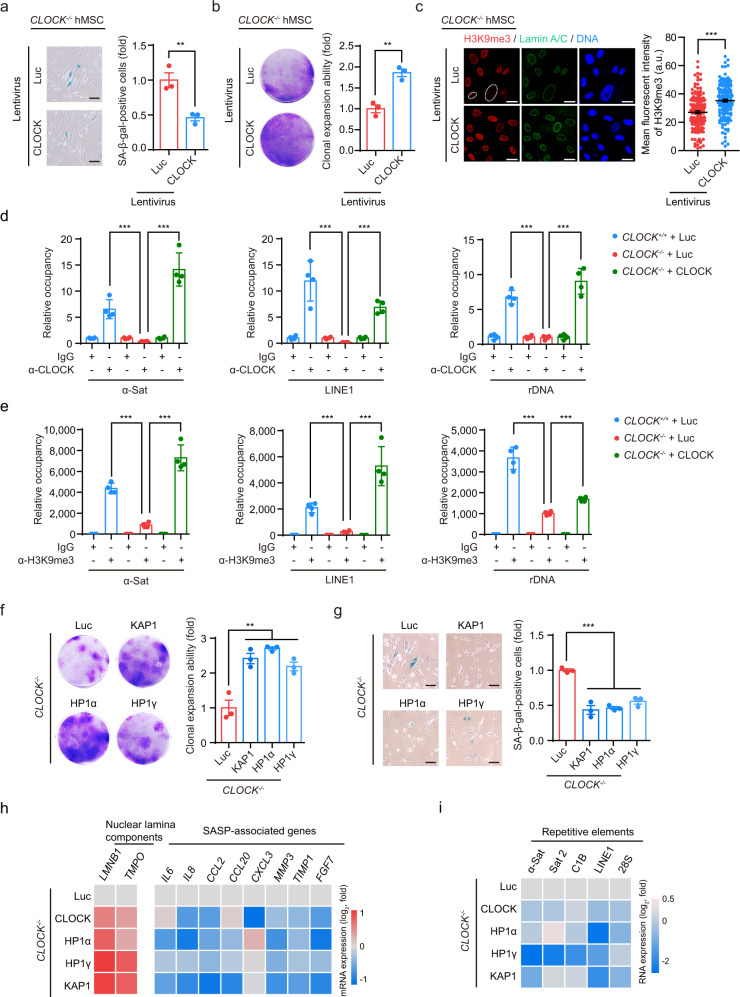
CLOCK–heterochromatin axis protects hMSC against senescence. **a** SA-β-gal staining of *CLOCK*^*−/−*^ hMSCs transduced with lentiviruses expressing Luc or CLOCK. Data are presented as means ± SEM. *n* = 3 biological replicates. **P < 0.01 (two-tailed unpaired Student’s *t*-test). Scale bars, 100 μm. **b** Clonal expansion assay of *CLOCK*^*−/−*^ hMSCs transduced with lentiviruses expressing Luc or CLOCK. Data are presented as means ± SEM. *n* = 3 biological replicates. **P < 0.01 (two-tailed unpaired Student’s *t*-test). **c** Immunofluorescence analysis of the H3K9me3 intensity in *CLOCK*^*–/–*^ hMSCs transduced with Luc or CLOCK. Dashed lines indicate the nuclear boundaries of the cells with decreased H3K9me3 signals. Data are presented as means ± SEM. *n* = 150 cells from three biological replicates. ***P < 0.001 (two-tailed unpaired Student’s *t*-test). Scale bars, 25 μm. **d** Enrichment of CLOCK within α-Sat, LINE1, and rDNA regions, as measured by ChIP-qPCR, in *CLOCK*^*+/+*^ hMSCs transduced with Luc and *CLOCK*^*−/−*^ hMSCs transduced with Luc or CLOCK. Data are presented as means ± SD. *n* = 4. ***P < 0.001 (two-tailed unpaired Student’s *t*-test). Data are representative of two independent experiments. **e** Enrichment of H3K9me3 within α-Sat, LINE1, and rDNA regions, as measured by ChIP-qPCR, in *CLOCK*^*+/+*^ hMSCs transduced with Luc and *CLOCK*^*−/−*^ hMSCs transduced with Luc or CLOCK. Data are presented as means ± SD. *n* = 4. ***P < 0.001 (two-tailed unpaired Student’s *t*-test). Data are representative of two independent experiments. **f** Clonal expansion assay of *CLOCK*^*−/−*^ hMSCs transduced with lentiviruses expressing Luc, KAP1, HP1α, or HP1γ. Data are presented as means ± SEM. *n* = 3 biological replicates. **P < 0.01 (two-tailed unpaired Student’s *t*-test). **g** SA-β-gal staining of *CLOCK*^*−/−*^ hMSCs transduced with lentiviruses expressing Luc, KAP1, HP1α, or HP1γ. Data are presented as means ± SEM. *n* = 3 biological replicates. ***P < 0.001 (two-tailed unpaired Student’s *t*-test). Scale bars, 100 μm. **h** Heatmaps showing the relative mRNA levels of nuclear lamina components and SASP-associated genes in *CLOCK*^*−/−*^ hMSCs transduced with lentiviruses expressing Luc, KAP1, HP1α, or HP1γ. The average expression levels of the indicated genes in each cell type were normalized to those in *CLOCK*^*−/−*^ hMSCs transduced with Luc. Data are representative of two independent experiments. **i** Heatmap showing the relative transcript levels of repetitive elements in *CLOCK*^*−/−*^ hMSCs transduced with Luc, KAP1, HP1α, or HP1γ. The average expression levels of the indicated repetitive elements in each cell type were normalized to those in *CLOCK*^*−/−*^ hMSCs transduced with Luc. Data are representative of two independent experiments.

Previous studies have suggested that Lamin B1, LBR and Emerin interact with heterochromatin components and facilitate the organization of lamina-associated domains (LADs) at the nuclear periphery.^57^ Consistent with this observation, nuclear enlargement and heterochromatin loss, characteristic of decreased expression levels of the nuclear lamina protein LBR and heterochromatin-associated proteins such as KAP1, HP1α and HP1γ, were observed in *CLOCK*^*−/−*^ hMSCs (Fig. 2f–i; Supplementary information, Fig. S3d). To investigate whether CLOCK was involved in heterochromatin maintenance, especially at LADs, we performed DNA adenine methyltransferase identification (DamID) assays in *CLOCK*^*+/+*^ and *CLOCK*^*−/−*^ hMSCs.^58^ The deficiency of CLOCK in hMSCs decreased the DamID signal intensity and coverage of LADs across the genome (Fig. 3a; Supplementary information, Fig. S4a–d). The violin plot further revealed LAD loss at repetitive genomic sequences, including centromeric satellite, LINE1, and rDNA loci (Fig. 3b). ChIP-sequencing (ChIP-seq) and ChIP-qPCR analysis revealed the genome-wide loss of the constitutive heterochromatin mark H3K9me3, especially within the LAD-specific heterochromatin regions (H3K9me3 mountains), in *CLOCK*^*–/–*^ hMSCs (Fig. 3c–f; Supplementary information, Fig. S4e–h). Consistent with the decreases in LADs and H3K9me3 in *CLOCK*^*–/–*^ hMSCs, the assay for transposase-accessible chromatin with sequencing (ATAC-seq) results showed increased chromatin accessibility at intron and intergenic regions, including long terminal repeats (LTRs), LINEs, short interspersed nuclear elements (SINEs), satellite DNA and rDNA, in *CLOCK*^–/–^ hMSCs compared to *CLOCK*^+/+^ hMSCs (Fig. 3g–i; Supplementary information, Fig. S4i). Consistent with the changes in chromatin architecture, RNA sequencing (RNA-seq) of late-passage hMSCs identified that a set of genes related to chromatin condensation were downregulated in *CLOCK*^–/–^ hMSCs (Supplementary information, Fig. S5a–d and Table S4). Given that heterochromatin maintenance is required for transcriptional silencing of repetitive sequences,^39^ we next examined the transcription levels of these genomic elements. As predicted, transcription from repetitive elements, including α-Sat, LINE1 and rRNA, was markedly induced in *CLOCK*^*–/–*^ hMSCs (Fig. 3j). Senescence phenotypes and epigenetic defects observed in CLOCK-deficient hMSCs were rescued by reintroduction of exogenous CLOCK, as evidenced by the decreased percentages of SA-β-gal-positive cells, increased clonal expansion ability, and restored H3K9me3 occupancy at repetitive sequences (Fig. 4a–e). These observations indicate that CLOCK is a new heterochromatin stabilizer in hMSCs.

We next individually overexpressed the known heterochromatin stabilizers (KAP1, HP1α or HP1γ) in *CLOCK*^−^^*/−*^ hMSCs to determine whether CLOCK exerted its geroprotective effects via the maintenance of heterochromatin. We observed alleviation of senescence phenotypes, similar to the effects of CLOCK overexpression, as indicated by the decreased percentages of SA-β-gal-positive cells, increased clonal expansion ability, and downregulated expression of SASP-associated genes and transcription of repetitive genomic sequences (Fig. 4f–i). Taken together, these data support the notion that CLOCK regulates hMSC aging by maintaining heterochromatin organization via the interactions with the nuclear envelope and heterochromatin components.

### CLOCK antagonizes senescence independent of its transcriptional activity

**Fig. 5:**
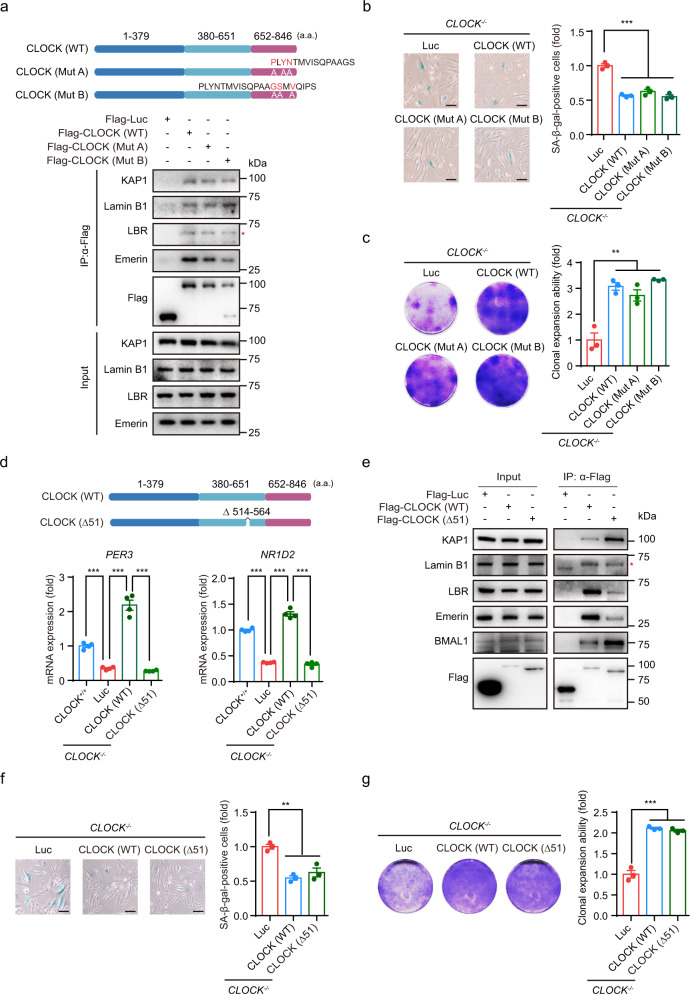
CLOCK counteracts hMSC senescence independent of its transcriptional activity. **a** Top, schematic diagram showing the mutation strategy for CLOCK. Bottom, Co-IP of KAP1 and nuclear lamina proteins with exogenous WT and mutant CLOCK proteins (Mut A and Mut B) in HEK293T cells. Data are representative of two independent experiments. The red asterisk indicates the LBR band pulled down by IP with Flag antibody. **b** SA-β-gal staining in *CLOCK*^*–/–*^ hMSCs transduced with lentiviruses expressing Luc, CLOCK (WT), CLOCK (Mut A), or CLOCK (Mut B). The percentage of SA-β-gal-positive cells in each cell type was normalized to that in *CLOCK*^*−/−*^ hMSCs transduced with Luc. Data are presented as means ± SEM. *n* = 3 biological replicates. ***P < 0.001 (two-tailed unpaired Student’s *t*-test). Scale bars, 100 μm. **c** Clonal expansion assay of *CLOCK*^*−/−*^ hMSCs transduced with lentiviruses expressing Luc, CLOCK (WT), CLOCK (Mut A), or CLOCK (Mut B). The clonal expansion ability of each cell type was normalized to that of *CLOCK*^*–/–*^ hMSCs transduced with Luc. Data are presented as means ± SEM. *n* = 3 biological replicates. **P < 0.01 (two-tailed unpaired Student’s *t*-test). **d** Top, schematic diagram showing the mutation strategy for the CLOCK Δ51 truncation. Bottom, relative mRNA levels of *PER3* and *NR1D2* in *CLOCK*^+/+^ hMSCs and *CLOCK*^*−/−*^ hMSCs transduced with lentiviruses expressing Luc, CLOCK (WT), or CLOCK (Δ51). Data are presented as means ± SEM. *n* = 4. ***P < 0.001 (two-tailed unpaired Student’s *t*-test). Data are representative of two independent experiments. **e** Co-IP of KAP1 and nuclear lamina proteins with exogenous CLOCK (WT) and CLOCK (Δ51) proteins in HEK293T cells. Data are representative of two independent experiments. The red asterisk indicates the band of Lamin B1. **f** SA-β-gal staining of *CLOCK*^*−/−*^ hMSCs transduced with lentiviruses expressing Luc, CLOCK (WT), or CLOCK (Δ51). The percentage of SA-β-gal-positive cells in each cell type was normalized to that in *CLOCK*^*–/–*^ hMSCs transduced with Luc. Data are presented as means ± SEM. *n* = 3 biological replicates. **P < 0.01 (two-tailed unpaired Student’s *t*-test). Scale bars, 100 μm. **g** Clonal expansion assay of *CLOCK*^*–/–*^ hMSCs transduced with lentiviruses expressing Luc, CLOCK (WT) and CLOCK (Δ51). The clonal expansion ability of each cell type was normalized to that of *CLOCK*^*–/–*^ hMSCs transduced with Luc. Data are presented as means ± SEM. *n* = 3 biological replicates. ***P < 0.001 (two-tailed unpaired Student’s *t*-test).

CLOCK has an intrinsic acetyltransferase domain that enables circadian chromatin remodeling by acetylating histones and nonhistone proteins,^59^ including its partner BMAL1, to promote the transcription of circadian clock-controlled genes.^60^ Thus, to determine whether the acetyltransferase activity of CLOCK was required for its regulatory role in hMSC senescence, we further generated two CLOCK mutants (Mut A and Mut B), which have been reported to exhibit reduced acetyltransferase activity due to the substitution of three amino acids (Supplementary information, Table S1).^59^ Subsequent Co-IP analysis revealed that CLOCK interacted with nuclear lamina proteins and KAP1 independent of its acetyltransferase region (Fig. 5a). In addition, introduction of exogenous CLOCK (Mut A) or CLOCK (Mut B) into *CLOCK*^–/–^ hMSCs effectively rescued senescence phenotypes, as evidenced by the decreased percentages of SA-β-gal-positive cells and increased cellular proliferation ability (Fig. 5b, c). These results suggest that CLOCK antagonizes cellular senescence independently of its acetyltransferase activity.

We then investigated whether the senescence-regulating activity of CLOCK required activation of its downstream target genes. To this end, we generated a human homolog (CLOCK Δ51, which lacks a.a. 514–564) of a previously reported mouse transcriptional activity-inactive mutant that encoded a CLOCK protein with a deletion of 51 amino acids in its transcriptional regulatory domain (Supplementary information, Table S1).^14,61^ While CLOCK Δ51 retained the ability to interact with nuclear lamina and heterochromatin-associated proteins, ectopic expression of CLOCK Δ51 in *CLOCK*^*−/−*^ hMSCs failed to induce the transcription of CLOCK target genes, including *PER3* and *NR1D2* (Fig. 5d, e). Notably, similar to its wild-type (WT) counterpart, CLOCK Δ51 retained the ability to rescue cellular senescence in *CLOCK*^*−/−*^ hMSCs (Fig. 5f, g). Taken together, these data support the notion that CLOCK suppresses hMSC aging in a heterochromatin-dependent but transcriptional activity-independent manner.

### Overexpression of CLOCK rejuvenates aged hMSCs

**Fig. 6:**
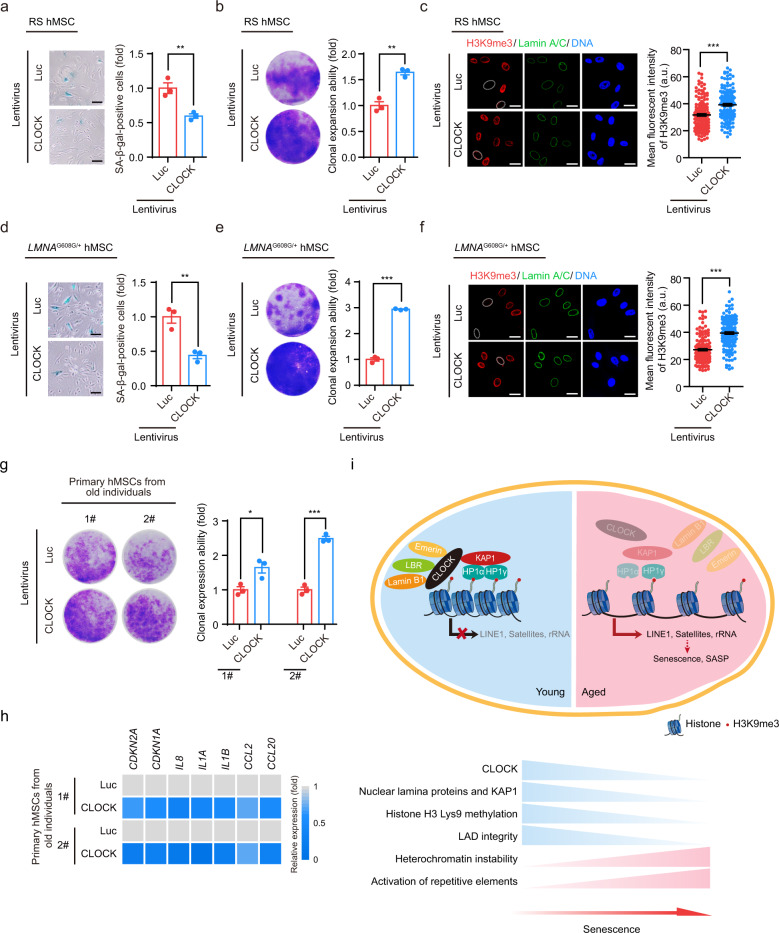
CLOCK overexpression attenuates hMSC aging. **a** SA-β-gal staining of replicative senescent (RS) *CLOCK*^*+/+*^ hMSCs transduced with lentiviruses expressing Luc or CLOCK. Data are presented as means ± SEM. *n* = 3 biological replicates. **P < 0.01 (two-tailed unpaired Student’s *t*-test). Scale bars, 100 μm. **b** Clonal expansion assay of RS *CLOCK*^*+/+*^ hMSCs transduced with lentiviruses expressing Luc or CLOCK. Data are presented as means ± SEM. *n* = 3 biological replicates. **P < 0.01 (two-tailed unpaired Student’s *t*-test). **c** H3K9me3 staining of RS *CLOCK*^*+/+*^ hMSCs transduced with lentiviruses expressing Luc or CLOCK. Dashed lines indicate the nuclear boundaries of cells with decreased H3K9me3 signals. Data are presented as means ± SEM. *n* = 150 cells from three biological replicates. ***P < 0.001 (two-tailed unpaired Student’s *t*-test). Scale bars, 25 μm. **d** SA-β-gal staining of HGPS-specific hMSCs transduced with lentiviruses expressing Luc or CLOCK. Data are presented as means ± SEM. *n* = 3 biological replicates. **P < 0.01 (two-tailed unpaired Student’s *t*-test). Scale bars, 100 μm. **e** Clonal expansion assay of HGPS-specific hMSCs transduced with lentiviruses expressing Luc or CLOCK. Data are presented as means ± SEM. *n* = 3 biological replicates. ***P < 0.001 (two-tailed unpaired Student’s *t*-test). **f** H3K9me3 staining of HGPS-specific hMSCs transduced with lentiviruses expressing Luc or CLOCK. Dashed lines indicate the nuclear boundaries of cells with decreased H3K9me3 signals. Data are presented as means ± SEM. *n* = 150 cells from three biological replicates. ***P < 0.001 (two-tailed unpaired Student’s *t*-test). Scale bars, 25 μm. **g** Clonal expansion assay of primary hMSCs that were derived from a 92-year-old individual and a 76-year-old individual and transduced with lentiviruses expressing Luc or CLOCK. Data are presented as means ± SEM. *n* = 3 biological replicates for two individuals. *P < 0.05; ***P < 0.001 (two-tailed unpaired Student’s *t*-test). **h** Heatmap showing the mRNA levels of the indicated genes in primary hMSCs transduced with lentiviruses expressing Luc or CLOCK. The average expression levels of the indicated genes of primary hMSCs transduced with lentiviruses expressing CLOCK were normalized to those of primary hMSCs transduced with lentiviruses expressing Luc. *n* = 4. **i** A constructive model describing the role of CLOCK in stabilizing heterochromatin during aging. In young hMSCs, CLOCK forms a complex with the heterochromatin-associated protein KAP1 and nuclear lamina proteins Lamin B1, LBR and Emerin. This complex tethers H3K9me3-enriched heterochromatin to nuclear periphery, repressing aberrant transcription of repetitive elements. In aged hMSCs, the complex is destabilized due to the downregulation of CLOCK and its binding partners, which results in heterochromatin instability and thereby transcription of repetitive elements, leading to cellular senescence and SASP.

The effect of CLOCK on antagonizing hMSC aging suggests that CLOCK overexpression may represent a new approach to rejuvenate aged hMSCs. Lentiviral vector-mediated ectopic expression of CLOCK alleviated senescence phenotypes in both replicative senescent hMSCs and HGPS-specific hMSCs, as demonstrated by the decreased percentages of SA-β-gal-positive cells, improved clonal expansion ability and increased levels of heterochromatin marks (Fig. 6a–f). Furthermore, the senescence-associated phenotypes in primary hMSCs isolated from a 92-year-old individual and a 76-year-old individual were attenuated by CLOCK overexpression (Fig. 6g, h). These data collectively suggest that overexpression of CLOCK is likely to reverse hMSC senescence (Fig. 6i).

### CLOCK-based gene therapy attenuates aging-associated articular degeneration in mice

**Fig. 7:**
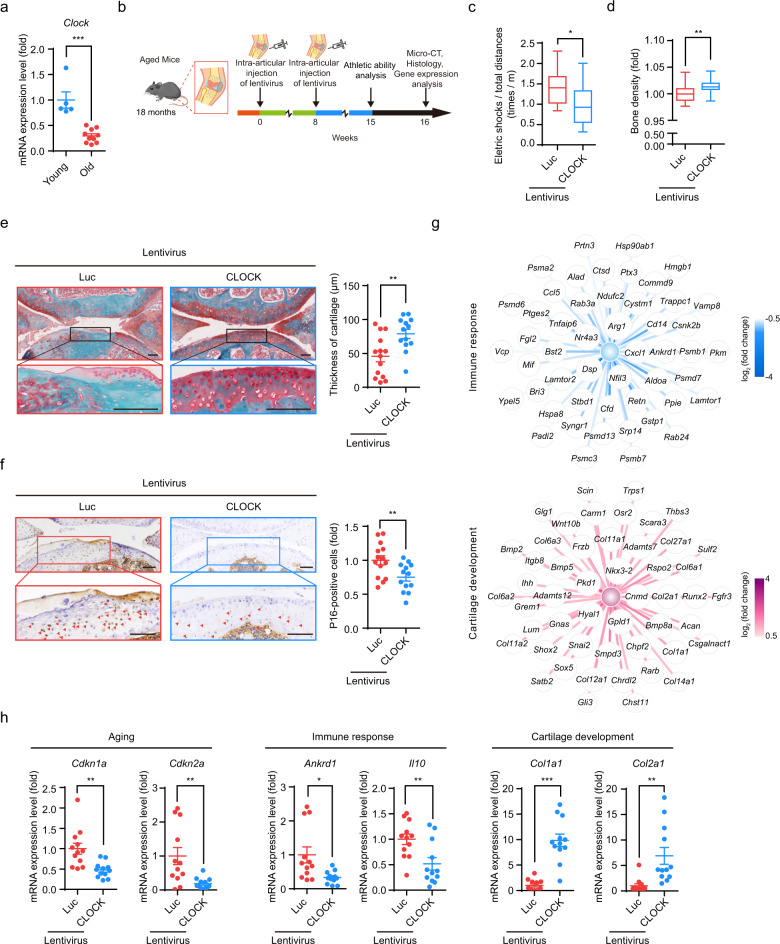
CLOCK gene therapy alleviates aging-associated osteoarthritis. **a** RT-qPCR analysis of *Clock* mRNA in the joints of young mice (*n* = 5) and old mice (*n* = 10). Data are presented as means ± SEM. ***P < 0.001 (two-tailed unpaired Student’s *t*-test). **b** Schematic of the time course for the experiments referenced in **b**–**h**. **c** Treadmill experiment with aged mice injected with lentiviruses expressing Luc (*n* = 14 mice) or CLOCK (*n* = 13 mice). Box plots indicate the median (center line inside the box), lower and upper quartiles (bounds of box), smallest and largest values (whiskers), *P < 0.05 (two-tailed unpaired Student’s *t-*test). **d** Bone density analysis of aged mice injected with lentiviruses expressing Luc (*n* = 14 mice) or CLOCK (*n* = 13 mice). Box plots indicate the median (center line inside the box), lower and upper quartiles (bounds of box), smallest and largest values (whiskers), **P < 0.01 (two-tailed unpaired Student’s *t*-test). **e** Representative images of safranin O-fast green staining of articular cartilage from aged mice injected with lentiviruses expressing Luc (*n* = 14 mice) or CLOCK (*n* = 13 mice). Quantitative data of articular cartilage thickness to the right are presented as means ± SEM. **P < 0.01 (two-tailed unpaired Student’s *t*-test). Scale bars, 100 μm. **f** Immunostaining for P16 in articular cartilage from aged mice injected with lentiviruses expressing Luc (*n* = 14 mice) or CLOCK (*n* = 13 mice). Quantitative data of the percentage of P16-positive cells to the right are presented as means ± SEM. **P < 0.01 (two-tailed unpaired Student’s *t*-test). Scale bars, 100 μm. **g** Network showing the expression levels of differentially expressed genes associated with “Immune response” and “Cartilage development” in the joints of aged mice injected with lentiviruses expressing Luc or CLOCK. Top 50 downregulated genes associated with “Immune response” and top 50 upregulated genes associated with “Cartilage development” are shown. **h** Representative RT-qPCR analysis of the indicated genes in the joints of aged mice injected with lentiviruses expressing Luc (*n* = 12 mice) or CLOCK (*n* = 12 mice). Data are presented as means ± SEM. *P < 0.05; **P < 0.01; ***P < 0.001 (two-tailed unpaired Student’s *t*-test).

We next evaluated whether lentiviral administration of CLOCK could alleviate aging-related syndromes. Articular degeneration is the most prevalent musculoskeletal disorder among the elderly and is a leading cause of disability during aging.^10^ Age-associated cartilage attrition has been at least partially attributed to the senescence of mesenchymal progenitor cells.^62–64^ Aging markers (such as *Cdkn1a* and *Cdkn2a*) and immune response-associated genes (such as *Il10* and *Cxcl10*) were upregulated whereas genes involved in cartilage development (such as *Col1a1* and *Col2a1*) were downregulated in the joints of old mice (15 months) when compared to those in the young mice (1 month) (Supplementary information, Fig. S6a). In addition, we observed that mRNA level of *Clock* in the joints of mice was downregulated during physiological aging (Fig. 7a). Accordingly, elimination or rejuvenation of senescent cells may be a possible therapeutic approach for treating age-related articular degeneration.^33,34,65–68^ To assess this possibility, lentiviral vectors encoding CLOCK or Luc (as the control) were injected into the knee joint articular cavities of 18-month-old (aged) mice and replenished at 8 weeks after the initial injection. Athletic ability was analyzed and microcomputed tomography (micro-CT) scanning was conducted at 15 and 16 weeks after the initial injection, respectively (Fig. 7b). Treadmill experiment showed that the athletic ability of aged mice was improved by injection of lentiviruses encoding CLOCK (Fig. 7c). Micro-CT and safranin O-fast green staining analyses revealed enhanced cartilage regeneration in the articular cavities of CLOCK-treated mice (Fig. 7d, e). Furthermore, reduced expression of the senescence marker P16 was observed on the articular cartilage surface in CLOCK-treated mice (Fig. 7f).^34,65^ RNA-seq of the stifle joints revealed downregulation of genes related to the immune response (such as *Il10*, *Nfil3*, and *Ankrd1*) and upregulation of genes involved in cartilage development (such as *Col1a1*, *Col2a1*, and *Runx2*) upon the administration of CLOCK lentiviral vectors (Fig. 7g; Supplementary information, Table S5). Consistent with these results, downregulation of the aging markers *Cdkn1a* and *Cdkn2a* and genes related to the immune response, as well as activation of genes involved in cartilage development, in the joints of CLOCK-treated mice was verified by RT-qPCR (Fig. 7h; Supplementary information, Fig. S6b). Meanwhile, the noncanonical role of CLOCK in the rejuvenation of senescent cells via heterochromatin stabilization was confirmed by increased H3K9me3 levels in the articular cartilage surface of aged mice administered with lentiviruses expressing CLOCK (Supplementary information, Fig. S6c). Moreover, RNA-seq analysis identified a set of genes related to “Condensed chromosome”, “Nuclear heterochromatin”, “DNA packaging complex” and “Chromatin” that were upregulated in the joints of aged mice injected with lentiviral CLOCK vectors (Supplementary information, Fig. S6d and Table S5). Taken together, these results support a geroprotective effect of CLOCK on the rejuvenation of aged cells, which may provide a therapeutic basis for promoting cartilage regeneration and alleviating the symptoms of age-related articular degeneration.

## Discussion

In the last few decades, CLOCK has been heavily studied as a circadian transcriptional activator.^14,61,69–71^ Here, we uncovered a noncanonical, circadian clock-independent role of CLOCK in maintaining heterochromatin organization and protecting hMSCs against senescence. We observed that CLOCK levels were decreased in replicative senescent, prematurely aged, and physiologically aged hMSCs. Mechanistically, CLOCK formed complexes with KAP1 and nuclear lamina proteins and stabilized heterochromatin at repetitive genomic sequences. Furthermore, our data revealed that overexpression of CLOCK rejuvenated hMSC senescence in vitro and alleviated aging-associated articular degeneration in vivo. Our findings expand the biological functions of CLOCK to an epigenetic regulator that stabilizes heterochromatin and provide a potential strategy for improving stem cell activity, tissue rejuvenation, and protecting against aging-related chronic conditions via the restoration of CLOCK expression.

Although previous studies predominantly utilized mouse models, we generated the first *CLOCK*-knockout hESCs and hMSCs by CRISPR/Cas9-mediated gene editing. CLOCK-deficient hESCs exhibited normal pluripotency, resembling their mESC counterparts.^72^ However, in contrast to *Clock*^*−/−*^ mESCs, which exhibited decreased proliferation and increased cell death,^72^ *CLOCK*^*−/−*^ hESCs displayed normal self-renewal kinetics. The difference may be due to the differences in species-specific properties and chromatin states between hESCs and mESCs.^73–77^ In contrast, CLOCK deficiency in hMSCs accelerated cellular senescence. For the first time, we identified that CLOCK formed protein complexes with the nuclear lamina and heterochromatin-associated proteins and bound to heterochromatin-enriched LADs in young hMSCs, thus stabilizing heterochromatin at repetitive sequence loci. Consistent with our findings, previous studies have shown in rat hepatocytes and neurons that CLOCK is localized not only at the transcriptionally active regions of chromatin but also at condensed chromatin.^78,79^

Similarly, another mouse circadian factor, PER complex (Per1 and Per2) has been reported to recruit the methyltransferase subunit HP1γ-Suv39h1 to establish an H3K9me3-associated repressive state, although the PER-HP1γ-Suv39h1 complex is localized mainly at the promoters of Per1 to repress its transcription.^80^ In addition, a recent study suggests the involvement of BMAL1 in the regulation of telomeric heterochromatin.^81^ Unlike BMAL1, which mainly maintains telomeric homeostasis, we found that CLOCK was enriched across diverse genomic repetitive sequences, including LINE1, satellite DNA, and rDNA, thus preventing aging-associated heterochromatin loss. Despite the different mechanisms, these studies collectively demonstrate the noncanonical roles of CLOCK and other core circadian proteins in regulating chromatin homeostasis. Whether there is a natural synergy between these circadian factors in heterochromatin stabilization during aging warrants further research. In this study, we found that CLOCK deficiency resulted in accelerated hMSC senescence accompanied by dysregulated molecular rhythms, as indicated by the decreased amplitudes or prolonged periods of PER3, DBP, NR1D1, and NR1D2 oscillations. Unlike epidermal and muscle stem cells in aged mice, which maintain robust core clock oscillation, a dampened molecular clock machinery was observed in late-passage hMSC (Supplementary information, Fig. S2e).^13^ The differences may be due to species- and cell type-specific regulation during aging. However, we excluded the possibility that the transcriptional activity of CLOCK on its target genes may be required for counteracting cellular senescence by showing that the transcriptionally inactive CLOCK mutant (CLOCK Δ51) and histone acetyltransferase activity-deficient CLOCK mutant retained the ability to stabilize heterochromatin and suppress senescence in hMSCs. Based on the findings that these mutants interacted with the nuclear lamina and heterochromatin, along with the observations made with the series of truncation mutants, we propose a model that CLOCK, especially its N-terminal domain, may participate in heterochromatin maintenance by functioning as a scaffold protein tethering heterochromatin components to the nuclear matrix, consistent with our recent discovery of a panel of new heterochromatin partners including DGCR8, WRN, SIRT7, and ZKSCAN3 as geroprotectors.^23,34,36,82^ Supporting our finding, another core circadian protein PER2 has also been found to function as a scaffold protein maintaining cellular homeostasis.^83^

The most exciting finding of this study may be the therapeutic potential of CLOCK expression vector in reversing cellular senescence and treating age-related disorders such as osteoarthritis. Aging is accompanied by stem cell exhaustion, and functional decay of articular mesenchymal progenitor cells has been identified as a causal factor for progressive articular degeneration.^62–64,67^ In particular, preventing cellular senescence via lentiviral administration of rejuvenating factors has been reported as a robust approach to treat age- or injury-associated articular degeneration.^33,34,65,84,85^ Our study provides strong evidence that gene therapy with CLOCK lentiviral expression vectors confers a rejuvenating effect on aged articular stem cells and promotes cartilage regeneration, ultimately resulting in alleviation of articular decay. Other beneficial effects of CLOCK, such as the promotion of cellular circadian clock homeostasis and chondrogenesis (Supplementary information, Fig. S2b, d), may also contribute to its therapeutic effect on age-related articular degeneration.^4,10^ Taken together, our findings provide new insights that link the circadian clock and epigenetic regulation with aging and emphasize the CLOCK–heterochromatin axis as a novel potential target for the future treatment of aging-associated degenerative disorders.

## Materials and methods

### Antibodies

For western blotting: anti-CLOCK (#5157, 1:1,000), anti-HP1α (#2616S, 1:1,000) and anti-HP1γ (#2619, 1:3,000) from Cell Signaling Technology; anti-KAP1 (Ab22553, 1:2,000), anti-Lamin B1 (Ab16048, 1:1,000) and anti-LBR (Ab32535, 1:1,000) from Abcam; anti-β-actin (sc-69879, 1:3,000) from Santa Cruz Biotechnology.

For immunostaining: anti-Ki67 (ZM0166, 1:500) from ZSGB-BIO; anti-γH2AX (05-636, 1:400) from Millipore; anti-53BP1 (A300-273A, 1:500) from Bethyl Laboratories; anti-FOXA2 (8186S, 1:100) from Cell Signaling Technology; anti-SMA (A5228, 1:100), and anti-TuJ1 (T2200, 1:100) from Sigma-Aldrich; anti-H3K9me3 (Ab8898, 1:500) and anti-NANOG (Ab21624, 1:200) from Abcam; anti-Lamin A/C (sc-376248, 1:500), anti-OCT3/4 (sc-5279, 1:200) and anti-SOX2 (sc-17320, 1:100) from Santa Cruz Biotechnology; anti-Aggrecan (AF1220, 1:100), anti-Osteocalcin (MAB1419, 1:100), and anti-FABP4 (AF3150, 1:100) from R&D Systems.

For flow cytometry: anti-CD73 (550257, 1:200), anti-CD90 (555595, 1:200), anti-CD44 (550989, 1:200), anti-HLA-ABC (560168, 1:100), anti-CD34 (555822, 1:200), anti-CD43 (560198, 1:200), anti-CD45 (555482, 1:200), anti-CD14 (555398, 1:200) and anti-CD19 (555415, 1:200) from BD Biosciences; anti-CD105 (17-1057-41, 1:200) and anti-PDPN (17-9381-42, 1:200) from eBioscience (San Diego, CA, USA); anti-CD29 (303004, 1:200), and anti-CD13 (301705, 1:200), anti-CD166 (343903, 1:200) and anti-CD164 (324805, 1:200) from Biolegend (San Diego, CA, USA).

### Cell culture

*CLOCK*^*+/+*^ hESCs (hESCs, line H9, WiCell Research Institute) and *CLOCK*^*−/−*^ hESCs were maintained on feeder layers that consisted of mitomycin C-inactivated mouse embryonic fibroblasts (MEFs) in hESC culture medium. The hESC culture medium contained DMEM/F12 (Thermo Fisher Scientific), 20% KnockOut Serum Replacement (Thermo Fisher Scientific), 0.1 mM nonessential amino acids (NEAAs, Thermo Fisher Scientific), 2 mM GlutaMAX (Thermo Fisher Scientific), 55 μM β-mercaptoethanol (Thermo Fisher Scientific), 1% penicillin/streptomycin (Thermo Fisher Scientific) and 10 ng/mL bFGF (Joint Protein Central, Incheon, Korea). Alternatively, hESCs were cultured on Matrigel (BD Biosciences, San Jose, CA, USA)-coated plates in mTeSR medium (STEMCELL Technologies, Vancouver, Canada).

Both hESC-derived hMSCs and primary hMSCs were maintained in MSC medium containing MEMα (Thermo Fisher Scientific) supplemented with 10% fetal bovine serum (FBS) (Cat# 10099-141, Lot# 1616964, Thermo Fisher Scientific), 0.1 mM NEAAs (Thermo Fisher Scientific), 1% penicillin/streptomycin (Thermo Fisher Scientific), and 1 ng/mL bFGF (Joint Protein Central, Incheon, Korea).

### CRISPR/Cas9-mediated gene editing in hESCs

CRISPR/Cas9-mediated gene editing was performed via previously described methods with some modifications.^33,34,65^ In brief, a guide RNA targeting exon 5 of *CLOCK* (*CLOCK*-gRNA) was cloned into a gRNA cloning vector (#41824, Addgene) (Supplementary information, Table S1). Upon treatment with a ROCK inhibitor Y-27632 (S1049, Selleck) for 24 h, hESCs (5 × 10^6^) were resuspended in 100 μL of Opti-MEM (Thermo Fisher Scientific) containing the plasmid cocktail, which consisted of the donor plasmid containing the homology arms, *CLOCK-*gRNA and hCas9 (#41815, Addgene), and electroporated in a 4D-Nucleofector system (Lonza). Cells were then seeded on MEF feeder cells. G418 (100 μg/mL, 11811023, Thermo Fisher Scientific) was added to initiate positive selection 2–4 days after electroporation. After 2 weeks of selection, G418-resistant clones were picked and transferred to a 96-well plate for further characterization and expansion. Genomic PCR and western blotting were used for genomic editing identification. Additionally, we removed the neomycin-resistance cassette in *CLOCK*^*−/−*^ hESCs via electroporation with the pCAG-FLpo-2A-puro vector. Three days after transfection, 1 μg/mL puromycin (Thermo Fisher Scientific) was used to enrich puromycin-resistant cells for 48 h. After 8–12 days of selection, the emerging colonies were picked and expanded for subsequent study.

### hMSC generation and characterization

hMSCs were differentiated from hESCs following a previously published protocol.^25,86,87^ In brief, hESCs were dissociated into embryoid bodies (EBs) and treated with differentiation medium (MEMα (Invitrogen) supplemented with 10% FBS (Cat# 10099-141,Lot# 1616964, Thermo Fisher Scientific), 0.1mM NEAAs (ThermoFisher Scientific), 10 ng/mL bFGF, 5 ng/mL TGFβ, and 1% penicillin/streptomycin (Gibco)) on Matrigel-coated plates for 10 days until 95% confluence. Next, the confluent MSC-like cells were digested and plated on Matrigel-coated plates treated with MSC culture medium containing MEMα (Invitrogen) supplemented with 10% FBS, 0.1 mM NEAAs, 1 ng/mL bFGF, and 1% penicillin/streptomycin (Gibco). Then, the confluent MSC-like cells were passaged to gelatin-coated plates. The hMSCs were purified with different antibodies corresponding to hMSC-specific markers (CD73, CD90, and CD105) by FACS. The CD73, CD90 and CD105 triple-positive cells were further characterized by surface antigen markers, including positive markers, such as CD44, CD166, CD29, HLA-ABC, and CD13, and negative markers, such as CD34, CD43, CD45, CD164, CD14, CD19, and PDPN. The functionality of hMSCs was further verified by differentiation towards chondrocytes, adipocytes and osteoblasts. The osteoblasts, chondrocytes and adipocytes derived from hMSCs were characterized by von Kossa staining (GMS 80045.3, GenMed Scientific Inc., USA) and osteocalcin staining (indicating osteogenesis), toluidine blue (T3260, Sigma) and aggrecan staining (indicating chondrogenesis), and Oil Red O (O1391, Sigma) staining (indicating adipogenesis) following the manufacturer’s instructions.

### Isolation and culture of primary hMSCs

Primary hMSCs were isolated from the gingiva of different individuals.^23,33,34,65^ The tissues separated from the gingiva were cut into small (1 mm^2^) pieces using scissors in TrypLE^™^ Express Enzyme (1 ×) plus Dispase IV and further incubated at 37 °C for 30 min. The digested tissue suspensions were neutralized by MSC culture medium and centrifuged at 200 × *g* for 5 min at room temperature. The resulting pellets were resuspended in MSC culture medium containing MEMα (Invitrogen) supplemented with 10% FBS (Gibco), 1 ng/mL bFGF, and 1% penicillin/streptomycin (Gibco) and plated onto gelatin-coated plates for the growth of primary hMSCs.

### Luciferase reporter assay

The *PER2-dLuc* plasmid was a kind gift from E.E. Zhang.^88^ Cells harboring *PER2-dLuc* were grown to confluence in 24-well culture plates and synchronized with 20 μM forskolin (S2449, Selleck) for 2 h. The medium was then replaced with MSC culture medium containing 0.25 mM luciferin (LUCK-1G, GoldBio). Cells were monitored in a LumiCycle luminometer at 37 °C for 5–7 days; the generated data were analyzed with LumiCycle Analysis software (Actimetrics). The data from the first 24-h cycle were excluded from our statistical analysis.

### In vitro hMSC synchronization and circadian analysis

hMSCs were plated on gelatin-coated plates with MSC medium until 95% confluence. For synchronization, cells were then treated with 20 μM forskolin for 2 h and re-stored in MSC medium after washing twice with PBS. Cells were collected starting from 24 h post-synchronization at 3-h intervals for 9 time points, followed by RNA extraction and RT-qPCR detection. The nonparametric test JTK_CYCLE was used for verifying circadian oscillations as previously described.^89^ Time course threads of hMSCs with both *p* and *q* values < 0.05 were considered rhythmic.

### Western blotting

Cells were lysed in SDS buffer containing 4% SDS and 100mM Tris-HCl. The protein concentration was quantified using a BCA quantification kit (Thermo Fisher Scientific), and ~20 μg of protein per sample was subjected to SDS-PAGE and electrotransferred to PVDF membranes (Millipore). Then, membranes were blocked with 5% milk, and incubated with primary antibodies and then with horseradish peroxidase (HRP)-conjugated secondary antibodies. Immunoreactive bands were visualized in a ChemiDoc XRS+ system (Bio-Rad). Statistical analyses were quantified by Image J software (NIH). Each group had three independent experiments. Statistical significances were assessed by a two-tailed unpaired Student’s *t*-test.

### Transmission electron microscopy

Late-passage (P9) *CLOCK*^*+/+*^ and *CLOCK*^*−/−*^ hMSCs were harvested enzymatically with TrypLE (Thermo Fisher Scientific) and centrifuged at 500 × *g* for 5 min at room temperature. The resulting pellets were fixed with 4% paraformaldehyde (PFA) in PBS (pH 7.4) on ice overnight. Cells were subsequently dehydrated in a graded series of ethanols, permeabilized and embedded in Lowicryl resin HM20. Sections (200 nm) were obtained and imaged with a Spirit transmission electron microscope (FEI Company) operating at 100 kV.

### Immunofluorescence staining

Cells seeded on coverslips (Thermo Fisher Scientific) were washed twice with PBS. Then, cells were fixed with 4% PFA for 30 min, permeabilized with 0.4% Triton X-100 in PBS for 30 min and blocked with 10% donkey serum in PBS (Jackson Immuno Research) for 1 h at room temperature. After blocking, cells were incubated with primary antibodies at 4 °C overnight. Subsequently, cells were washed three times with PBS, incubated with secondary antibodies at room temperature for 1 h and washed three times with PBS. Nuclei were stained with Hoechst 33342 (H3570, Thermo Fisher Scientific). Imaging was performed with a Leica SP5 confocal system. Statistical analysis of number, intensity and area of fluorescence signals were quantified by Image J software (NIH). Cells were collected from three biological replicates. Statistical significances were assessed by a two-tailed unpaired Student’s *t*-test. The 3D reconstruction of H3K9me3 staining as shown in Fig. 2f was performed by serial z-stack sectioning by 50-nm intervals at a conventional mode for up to 50 images with a Leica SP5 confocal system and further processed for 3D reconstruction by Imaris software (version 7.4.2) as previously described.^90^

### SA-β-gal staining assay

SA-β-gal staining was performed as described in previous studies.^33,34^ In brief, cells were fixed with fixation buffer (2% formaldehyde and 0.2% glutaraldehyde) for 5 min at room temperature. Then, fixed cells were stained with fresh staining solution at 37 °C overnight. Fields of view were randomly selected in each well, and the percentage of SA-β-gal-positive cells was determined using ImageJ software. Each group had three biological replicates. Statistical significances were assessed by a two-tailed unpaired Student’s *t*-test.

### Clonal expansion assay

A clonal expansion assay was conducted as previously reported.^34,65^ In brief, 6000 cells per well were seeded into 6-well plates and cultured for 9–12 days. Cells were washed twice with PBS, fixed with 4% PFA and stained with 0.2% crystal violet for 1 h at room temperature. Fields of view were scanned by a scanner and further measured by ImageJ software (NIH). Each group had three biological replicates. Statistical significances were assessed by a two-tailed unpaired Student’s *t*-test.

### Co-IP

HEK293T cells transfected with plasmids expressing Flag-Luc or Flag-CLOCK and hMSCs were lysed in CHAPS lysis buffer (120 mM NaCl, 0.3% CHAPS, 1 mM EDTA, 40 mM HEPES (pH 7.5), and complete protease inhibitor cocktail (Roche)) at 4 °C for 4 h and were then centrifuged at 14,500 × *g* at 4 °C for 40 min. For Co-IP with exogenous proteins, the supernatants were mixed with anti-Flag antibody-coupled beads (A2220, Sigma) and rotated overnight at 4 °C. For Co-IP with endogenous proteins, the supernatants were mixed with the indicated antibodies overnight at 4 °C with rotation and were then incubated with Protein A/G-PLUS Agarose beads (sc-2003, Santa Cruz) for another 4 h at 4 °C with rotation. The beads were washed six times with CHAPS buffer and then eluted with Flag peptides or by boiling in 1× SDS loading buffer for 10 min for western blotting.

### LC-MS/MS analysis and protein identification

The eluted proteins obtained by immunoprecipitation were subjected to 10% SDS-PAGE and stained with Coomassie brilliant blue (WB-0101, Beijing Dingguo Changsheng Biotechnology Co. Ltd). The gel bands containing the protein samples were excised, cut into small plugs, dehydrated (100% acetonitrile), reduced (10 mM DTT in 25 mM NH_4_HCO_3_ for 45 min at 56 °C) and alkylated (40 mM iodoacetamide in 25 mM NH_4_HCO_3_ for 45 min at room temperature in the dark). Next, the gel plugs were dried and digested with sequencing-grade modified trypsin (40 ng per band) in 25 mM NH_4_HCO_3_ overnight at 37 °C. Finally, formic acid to a 1% final concentration was used to terminate the enzymatic reaction. The resulting solution was then transferred to a sample vial for LC-MS/MS analysis.

The nanoLC-MS/MS experiments were performed on a Q Exactive mass spectrometer (Thermo Scientific) in data-dependent mode, which allowed MS data acquisition at a high resolution of 70,000 (*m/z* 200) across an *m/z* range of 300–1,600. The raw data from Q Exactive analysis were analyzed with Proteome Discovery (version 1.4) using the Sequest HT search engine for protein identification and Percolator for false discovery rate (FDR) analysis. The data were searched against the UniProt human protein database (updated on 06-2013). FDR analysis was performed with Percolator, and FDR < 1% was set as the threshold for protein identification. The peptide confidence was set to high for peptide filtering.

### RT-qPCR and RNA-Seq

Total RNA was extracted from cultured human cells or mouse joints using TRIzol (Thermo Fisher Scientific), and genomic DNA was removed using a DNA-free kit (Thermo Fisher Scientific). cDNA was generated with the Go Script Reverse Transcription System (Promega). RT-qPCR was performed using qPCR Mix (Toyobo) in a CFX384 Real-Time system (Bio-Rad). Each group had four well replicates. Statistical significances were assessed by a two-tailed unpaired Student’s *t*-test. All RT-qPCR experiments were performed with at least three experimental replicates and independently repeated for at least two times. For mouse joint RNA-seq, knee joint RNA samples from the same treatment group of aged mice injected with lentiviruses expressing Luc (*n* = 12 mice) or CLOCK (*n* = 12 mice) were mixed equally in mass, and RNA-seq was performed with two technical replicates. For hMSC RNA-seq, two biological replicates were examined. A sequencing library was constructed using a NEBNext Ultra RNA Library Prep Kit for Illumina following the manufacturer’s protocol. The generated libraries were sequenced on Illumina HiSeq X-Ten platforms with paired-end sequencing with 150-bp read lengths. Quality control and sequencing were performed by NoVo gene Bioinformatics Technology. Primers used for qPCR were shown in Supplementary information, Table S2.

### RNA-seq data processing

The RNA-seq data processing pipeline has been reported previously.^34^ Raw paired-end reads were trimmed in Trim Galore software (version 0.4.5) (https://github.com/FelixKrueger/TrimGalore). Clean reads were mapped to the UCSC human hg19 genome or mouse mm10 genome using hisat2 (version 2.0.4).^91^ The sam files were converted to bam files by SAMtools with the parameter “-S -b -q 10”.^92^ Then, read counts were calculated by HTSeq (version 0.11.0), and only high-quality mapped reads (mapping quality > 20) were retained.^93^ The Fragments Per Kilobase per Million (FPKM) value for each gene was calculated using StringTie (version 1.2.3).^94^ Differentially expressed genes (DEGs) were calculated using DESeq2 package (version 1.22.2) with the cutoff “*q* value (adjusted *p* value, padj) < 0.05 and |log_2_ (fold change)| > 1 for hMSCs or |log_2_ (fold change)| > 0.5 for mouse joints”. Gene Ontology (GO) enrichment analysis was conducted with ToppGene.^95^ Gene set enrichment analysis was conducted by GSEA (version 2.2.4). SASP gene set was obtained from a previous study.^42^

### DamID-seq

pLgw V5-EcoDam and pLgw EcoDam-V5-EMD were gifts from Prof. Bas van Steensel (The Netherlands Cancer Institute (NKI)). DamID-seq was performed as described,^58^ with modifications. In brief, the Dam and Dam-EMD lentiviruses generated from HEK293T cells were concentrated by ultracentrifugation at 19,400× *g* for 2.5 h. The virus pellets were resuspended in PBS. *CLOCK*^*+/+*^ and *CLOCK*^*−/−*^ hMSCs were seeded in six-well dishes at 2 × 10^5^ cells per well. Each group had three biological replicates. The next day, the culture medium was replaced with 2 mL of fresh culture medium containing either Dam or Dam-EMD lentivirus. At 72 h after transduction, cells were harvested, and genomic DNA was isolated using a DNeasy Blood & Tissue Kit (69504, Qiagen). *Dpn*I (R0176S, New England Biolabs) digestion, adapter ligation, *Dpn*II (R0543S, New England Biolabs) digestion, PCR amplification and purification were performed as previously described.^58^ For adapter trimming, the amplified DNA was sonicated and digested with *Alw*I (R0513S, New England Biolabs). The DNA library was constructed using a NEBNext Ultra DNA Library Prep Kit for Illumina (E7370S, New England Biolabs). The libraries were pooled and subjected to paired-end sequencing with 150-bp read lengths on Illumina NovaSeq sequencers.

### DamID-seq data processing

Raw reads of Dam and Dam-EMD data for *CLOCK*^*+/+*^ and *CLOCK*^*−/−*^ hMSCs were trimmed in Trim Galore software (version 0.4.5) (https://github.com/FelixKrueger/TrimGalore). Trimmed reads were mapped to the UCSC human hg19 genome using Bowtie 2 (version 2.2.9).^96^ PCR duplicates were removed with the MarkDuplicates.jar program in Picard tools. Then, reads were sorted with SAMtools (version 1.6). To minimize the effect of sequencing bias and depth, processed reads from three replicates for each sample were merged. Then, the same number (110 million) of high-quality reads for each cell type were randomly selected for downstream analysis. To visualize the DamID signals, we calculated the log_2_ ratio of the Reads Per Kilobase per Million mapped reads (RPKM) of the Dam-EMD and Dam signals (log_2_ (Dam-EMD/Dam)) in *CLOCK*^*+/+*^ and *CLOCK*^*−/−*^ hMSCs for each 10-bp bin using the bamCompare program in deepTools (version 2.5.4-2-5ee467f) software.

For identification of LAD regions in *CLOCK*^*+/+*^ and *CLOCK*^*−/−*^ hMSCs, we first calculated the DamID signals (log_2_ ratio of the RPKM of the Dam-EMD and Dam signals (log_2_ (Dam-EMD/Dam)) in *CLOCK*^*+/+*^ and *CLOCK*^*−/−*^ hMSCs for each 2-kb bin using the bamCompare program in deepTools (version 2.5.4-2-5ee467f) software. Then, we implemented the R package for hidden Markov models with t emissions (HMMt) (version 0.1), to identify LADs (https://github.com/dinovski/asDamID/blob/master/scripts/hmmt_functions.R).

To compare the DamID signals in LAD regions between *CLOCK*^*+/+*^ and *CLOCK*^*−/−*^ hMSCs, we merged LAD regions identified in *CLOCK*^*+/+*^ and *CLOCK*^*−/−*^ hMSCs into union ones and calculated the relative DamID signal (DamID signal in *CLOCK*^*−/−*^ hMSCs minus DamID signal in *CLOCK*^*+/+*^ hMSCs) for each union LAD region. Then, the relative DamID signals for LAD regions were plotted using R package RIdeogram (version 0.2.2), as shown in Supplementary information, Fig. S4d.^97^

### ChIP-qPCR and ChIP-seq

In brief, 1 × 10^6^ *CLOCK*^*+/+*^ and *CLOCK*^*−/−*^ hMSCs were crosslinked in 1% (vol/vol) formaldehyde in PBS for 8 min at room temperature, and the reaction was terminated by incubation with 125 mM glycine for 5 min at room temperature. Samples were then lysed on ice for another 10 min. After sonication in Covaris S220 focused-ultrasonicator (Covaris) and centrifugation for 10 min at 12,000 × *g* at 4 °C, supernatants were incubated overnight at 4 °C with Protein A Dynabeads (Thermo Fisher Scientific, 10004D) conjugated with an anti-CLOCK antibody, an anti-H3K9me3 antibody or rabbit IgG. Subsequently, elution and reverse crosslinking were performed at 68 °C for 2 h in a thermomixer. Then, DNA was isolated via phenol-chloroform-isoamyl alcohol extraction and ethanol precipitation. The purified DNA was subjected to qPCR for the evaluation of CLOCK or H3K9me3 occupation at repetitive sequences. The enriched fragments were constructed into libraries without the incorporation of spike-in controls via KAPA Hyper Prep Kits with PCR Library Amplification/Illumina Series (KK8504, New England Biolabs) following the manufacturer’s instructions. All of the experiments were performed at least two times with similar results. Statistical significances were assessed by a two-tailed unpaired Student’s *t*-test.

### ChIP-seq data processing

For the processing of H3K9me3 ChIP-seq data, raw reads were trimmed with Trim Galore software (version 0.4.5) (https://github.com/FelixKrueger/TrimGalore). Trimmed reads were mapped to the UCSC human hg19 genome using Bowtie 2 (version 2.2.9).^96^ Duplicated reads were removed with the MarkDuplicates.jar program in Picard tools. Then, reads were sorted with SAMtools (version 1.6). To minimize the effect of sequencing bias and depth, processed reads from three replicates for each sample were merged. Then, the same number (130 million) of high-quality reads for each cell type were randomly selected for downstream analysis. To visualize the ChIP-seq signals, we calculated the normalized read counts by determining the RPKM values for each 10-bp bin. For H3K9me3 peak calling, SICER (version V1.1) was used with the parameter “-w 200 -g 3”.^98^ Only called H3K9me3 peaks with a cutoff FDR of 1% or higher were retained.

For the identification of “H3K9me3 mountains”, the H3K9me3 signal in counts per million (CPM) in each H3K9me3 peak was calculated. We then ranked the H3K9me3 peaks in the order of increasing H3K9me3 signal and plotted the H3K9me3 ChIP-seq occupancy in *CLOCK*^*+/+*^ and *CLOCK*^−/−^ hMSCs, as shown in Supplementary information, Fig. S4f. These plots showed a clear point where the H3K9me3 occupancy signal began increasing rapidly. Then, the inflection points of these curves were determined. We further defined H3K9me3 peaks above the inflection points as “H3K9me3 mountains” and H3K9me3 peaks below those points as typical H3K9me3 peaks.

### ATAC-seq

ATAC-seq was performed as described previously.^99^ In brief, a total of 50,000 cells were washed twice with PBS and resuspended in 50 μL of lysis buffer (10 mM Tris-HCl (pH 7.4), 10 mM NaCl, 3 mM MgCl_2_, 0.1% (v/v) Nonidet P40 substitute). The suspension of nuclei was then centrifuged at 500 × *g* for 10 min at 4 °C, followed by the addition of 50 μL of transposition reaction mix (10 μL of 5 × TTBL buffer, 4 μL of TTE mix and 36 μL of nuclease-free H_2_O) from a TruePrep DNA Library Prep Kit V2 for Illumina (Vazyme Biotech). Samples were then incubated at 37 °C for 30 min. The libraries were then amplified and purified using the TruePrep DNA Library Prep Kit V2 for Illumina (Vazyme Biotech). The library quality was assessed using a fragment analyzer. Finally, libraries were sequenced on Illumina HiSeq X-Ten platforms with paired-end sequencing with 150-bp read lengths.

### ATAC-seq data processing

For the processing of ATAC-seq data, raw reads were trimmed with Trim Galore software (version 0.4.5) (https://github.com/FelixKrueger/TrimGalore). Trimmed reads were mapped to the UCSC human hg19 genome using Bowtie 2 (version 2.2.9) with the parameter “-X 2000 -N 1 -L 25 –no-mixed –no-discordant -t”.^96^ Duplicate reads were removed with the MarkDuplicates.jar program in Picard tools. Then, reads were sorted with SAMtools (version 1.6) software. Then, processed reads from three replicates for each sample were merged. To visualize the ATAC signals, we extended each read by 250 bp and normalized the read counts by the RPKM value for each 10-bp bin. For ATAC peak calling, MACS2 (version 2.1.1.20160309) was used with the parameter “–nomodel –shift 0 –extsize 250 –call-summits”.^100^ Only called ATAC peaks with *q* values < 0.01 were retained. ATAC peaks identified in *CLOCK*^*+/+*^ but not in *CLOCK*^*−/−*^ hMSCs were defined as “Closed” ATAC peaks; ATAC peaks identified in *CLOCK*^*−/−*^ but not in *CLOCK*^*+/+*^ hMSCs were defined as “Opened” ATAC peaks. Estimation of the genomic distribution of ATAC peaks used annotatePeaks.pl program in homer software.^101^

### Assessment of the reproducibility of the sequencing data

To evaluate the reproducibility of the H3K9me3 ChIP-seq and ATAC-seq data, the Euclidean distance between replicates was calculated as follows: reads were counted and normalized by the RPKM at a 2-kb bin size. Then, the Euclidean distance was calculated in R (version 3.5.1) to evaluate the reproducibility. Lower Euclidean distances mean higher correlations. To evaluate the reproducibility of DamID-seq data, principal component analysis (PCA) was conducted in R (version 3.5.1). To evaluate the reproducibility of RNA-seq data, scatter plots were drawn based on the regularized logarithm (rlog)-normalized read count with DESeq2, and the Pearson correlation coefficient between replicates was calculated.

### Animal experiments

For teratoma formation analysis, NOD/SCID mice (6–8 weeks old, purchased from Beijing Vital River Laboratory Animal Technologies Co. Ltd) were injected with 3 × 10^6^ *CLOCK*^*+/+*^ or *CLOCK*^−/−^ hESCs in a Matrigel: mTeSR (1:4) solution. Teratomas were harvested 8–12 weeks after injection for further analysis.

For hMSC transplantation assays, 1 × 10^6^ *CLOCK*^*+/+*^ or *CLOCK*^*−/−*^ hMSCs transduced with lentiviruses expressing Luc were injected into the TA muscle of nude mice (6–8 weeks old). Mice were then treated with D-luciferin (LUCK-1G, GoldBio) and imaged with an IVIS Spectrum imaging system (Xenogen, Caliper, Waltham, MA, USA). Bioluminescence images were acquired in “auto” mode. Each group had six biological replicates. Statistical significances were assessed by a two-tailed unpaired Student’s *t*-test.

For the aging-associated *Clock* level detection, mice of different ages (young, 1 month, *n* = 5 mice; old, 15 months, *n* = 10 mice) were euthanized and the joints were collected for RT-qPCR analysis. Statistical significances were assessed by a two-tailed unpaired Student’s *t*-test.

To evaluate whether lentiviral administration of CLOCK could alleviate aging-related syndromes, lentiviruses expressing Luc (*n* = 14 mice) or CLOCK (*n* = 13 mice) were injected into the articular cavities of 18-month-old mice (purchased from SPF (Beijing) Biotechnology) and replenished after 8 weeks of injection. Treadmill experiment was conducted at time of 15 weeks after the first injection of lentiviruses expressing Luc or CLOCK. In detail, mice were trained on a treadmill (SA101, SANS) at an incline of 5° over 3 days for 20 min each day, with the speed accelerated from 0 to 20 m/min. On the test day, the mice ran on the treadmill with an initial speed of 0 m/min, and then the speed was increased by 2 m/min to 20 m/min. The whole running time was set at 20 min. The frequency of mild electrical shock stimulus was recorded and analyzed (Luc, *n* = 14 mice; CLOCK, *n* = 13 mice). Micro-CT scanning was performed at time of 16 weeks after the first injection (Luc, *n* = 14 mice; CLOCK, *n* = 13 mice). Mice were then euthanized, and the joints were collected for histological assessment (Luc, *n* = 14 mice; CLOCK, *n* = 13 mice) and mRNA quantification (Luc, *n* = 12 mice; CLOCK, *n* = 12 mice). Statistical significances were assessed by a two-tailed unpaired Student’s *t*-test.

### Histology and immunohistochemistry

For histological analysis, harvested mouse joints were fixed with 4% PFA for 2 days and embedded in paraffin after decalcification for 14–21 days. Sections (5 μm) were stained with fast green FCF (0.02%) and safranin O (0.1%) according to the manufacturer’s instructions.

Immunohistochemical staining was performed using the DAB staining method as previously described, with some modifications.^33,34,102^ First, slides were deparaffinized and rehydrated using xylene and different concentrations of alcohol. Antigen retrieval was performed using Trypsin (ZLI-9010, ZSGB-BIO) digestion for 20 min at room temperature. Hydrogen peroxide (3%) was used to block endogenous peroxidase activity by incubating for 10 min at room temperature. Slides were then blocked with 10% donkey serum for 1 h at room temperature and incubated with an anti-P16 antibody (Ab54210, Abcam, 1:200) at 4 °C overnight and with a secondary antibody (PV-6002, ZSGB-BIO) for 1 h at room temperature before DAB staining (ZLI-9017, ZSGB-BIO).

### Ethical declarations

The experiments involved in this study followed the Principles for the Application Format for Ethical Approval for Research Involving Animals and were approved in advance by the Institutional Animal care and Use Committee of the Institute of Zoology (Chinese Academy of Sciences). Anaesthetization of mice was conducted with isoflurane and euthanization was performed with CO_2_ followed by cervical dislocation.

### Statistical analysis

Statistical analyses were performed using Graph-Pad Prism Software. Data are presented as means ± SEM or SD. Comparisons were performed with two-tailed unpaired Student’s *t*-test. *P* value (*p*) < 0.05 was defined as statistically significant.

## Supplementary information

Figure S1

Figure S2

Figure S3

Figure S4

Figure S5

Figure S6

Table S1

Table S2

Table S3

Table S4

Table S5

## Data Availability

The sequencing data obtained in this study were uploaded to the Gene Expression Omnibus (GEO) database under the accession number GSE145019.

## Supplementary information

**Supplementary information** accompanies for this paper at 10.1038/s41422-020-0385-7.
