# Supplementary material for: Stabilization of heterochromatin by CLOCK promotes stem cell rejuvenation and cartilage regeneration
Source: Cell Res. 2020 Jul 31;31(2):187–205. doi: 10.1038/s41422-020-0385-7 (PMC8027439; doi:10.1038/s41422-020-0385-7)
Supplement: Supplementary file 1 — Figure S1 [file 41422_2020_385_MOESM1_ESM.pdf]

Figure S1

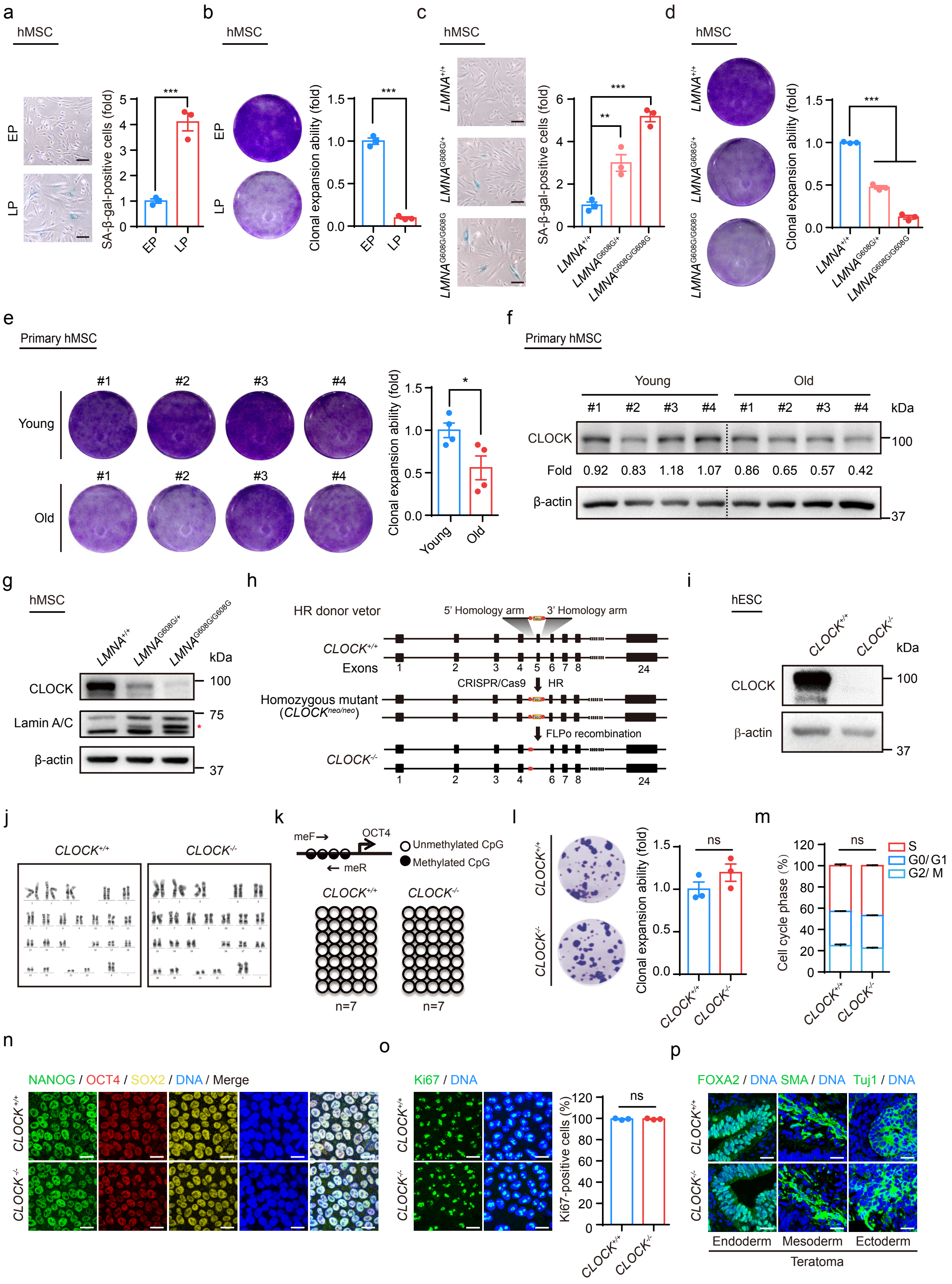

a
hMSC
EP
LP
SA-β-gal-positive cells (fold)
***
b
hMSC
Clonal expansion ability (fold)
c
hMSC
LMNA+/+
LMNAG608G/+
LMNAG608G/G608G
d
e
Primary hMSC
Young
Old
#1
#2
#3
#4
f
Primary hMSC
CLOCK
Fold 0.92 0.83 1.18 1.07 0.86 0.65 0.57 0.42
β-actin
kDa
100
37
g
hMSC
Lamin A/C
75
h
HR donor vetor
5' Homology arm
3' Homology arm
CLOCK+/+
Exons
CRISPR/Cas9
HR
Homozygous mutant (CLOCKneo/neo)
FLPo recombination
CLOCK-/-
i
hESC
j
k
meF
OCT4
meR
Unmethylated CpG
Methylated CpG
n=7
l
ns
m
Cell cycle phase (%)
S
G0/ G1
G2/ M
n
NANOG / OCT4 / SOX2 / DNA / Merge
o
Ki67 / DNA
Ki67-positive cells (%)
p
FOXA2 / DNA SMA / DNA Tuj1 / DNA
Endoderm
Mesoderm
Ectoderm
Teratoma

Supplementary information, Fig. S1 Characterization of senescent hMSCs and generation of CLOCK-deficient hESCs. a SA-β-gal staining of EP (P4) and LP (P13) *CLOCK*$^{+/+}$ hMSCs. Data are presented as means ± SEM. $n = 3$ biological replicates. ***$p < 0.001$ (Two-tailed unpaired Student's *t*-test). Scale bars, 100 μm. **b** Clonal expansion assay of EP (P4) and LP (P12) *CLOCK*$^{+/+}$ hMSCs. Data are presented as means ± SEM. $n = 3$ biological replicates. ***$p < 0.001$ (Two-tailed unpaired Student's *t*-test). **c** SA-β-gal staining of HGPS-specific hMSCs. Data are presented as means ± SEM. $n = 3$ biological replicates. **$p < 0.01$; ***$p < 0.001$ (Two-tailed unpaired Student's *t*-test). Scale bars, 100 μm. **d** Clonal expansion assay of HGPS-specific hMSCs. Data are presented as means ± SEM. $n = 3$ biological replicates. ***$p < 0.001$ (Two-tailed unpaired Student's *t*-test). **e** Clonal expansion assay of primary hMSCs derived from young and old individuals. Data are presented as means ± SEM. $n = 4$ biological replicates. *$p < 0.05$ (Two-tailed unpaired Student's *t*-test). **f** Representative image of western blot analysis of CLOCK in young and old human primary MSCs. β-actin was used as the loading control. $n = 4$ biological replicates. **g** Representative image of western blot analysis of CLOCK in HGPS-specific (*LMNA*$^{G608G/+}$ and *LMNA*$^{G608G/G608G}$) hMSCs. β-actin was used as the loading control. * represents the band of progerin. **h** Schematic showing the gene editing strategy for *CLOCK* (exon 5) using CRISPR/Cas9-mediated HR in hESCs. **i** Western blot analysis of CLOCK in both *CLOCK*$^{+/+}$ and *CLOCK*$^{-/-}$ hESCs. β-actin was used as the loading control. Data are representative of three independent experiments. **j** G-banding karyotyping of *CLOCK*$^{+/+}$ and *CLOCK*$^{-/-}$ hESCs. **k** DNA methylation analysis at the *OCT4* promoter in *CLOCK*$^{+/+}$ and *CLOCK*$^{-/-}$ hESCs. **l** Clonal expansion assay of *CLOCK*$^{+/+}$ and *CLOCK*$^{-/-}$ hESCs. Data are presented as means ± SEM. $n = 3$ biological replicates. ns, nonsignificant (Two-tailed unpaired Student's *t*-test). **m** Cell cycle analysis of *CLOCK*$^{+/+}$ and *CLOCK*$^{-/-}$ hESCs. Data are presented as means ± SEM. $n = 3$ biological replicates. ns, nonsignificant (Two-tailed unpaired Student's *t*-test). **n** Immunofluorescence analysis of the pluripotency markers NANOG, OCT4, and SOX2 in *CLOCK*$^{+/+}$ and *CLOCK*$^{-/-}$ hESCs. Scale bars, 25 μm. **o** Immunofluorescence analysis of Ki67 in *CLOCK*$^{+/+}$ and *CLOCK*$^{-/-}$ hESCs. Data are presented as means ± SEM. $n = 3$ biological replicates. ns, nonsignificant (Two-tailed unpaired Student's *t*-test). Scale bars, 25 μm. **p** Immunofluorescence analysis of representative markers of the three germ layers in teratomas derived from *CLOCK*$^{+/+}$ and *CLOCK*$^{-/-}$ hESCs. Scale bars, 25 μm.
