# Supplementary material for: Stabilization of heterochromatin by CLOCK promotes stem cell rejuvenation and cartilage regeneration
Source: Cell Res. 2020 Jul 31;31(2):187–205. doi: 10.1038/s41422-020-0385-7 (PMC8027439; doi:10.1038/s41422-020-0385-7)
Supplement: Supplementary file 2 — Figure S2 [file 41422_2020_385_MOESM2_ESM.pdf]

Figure S2

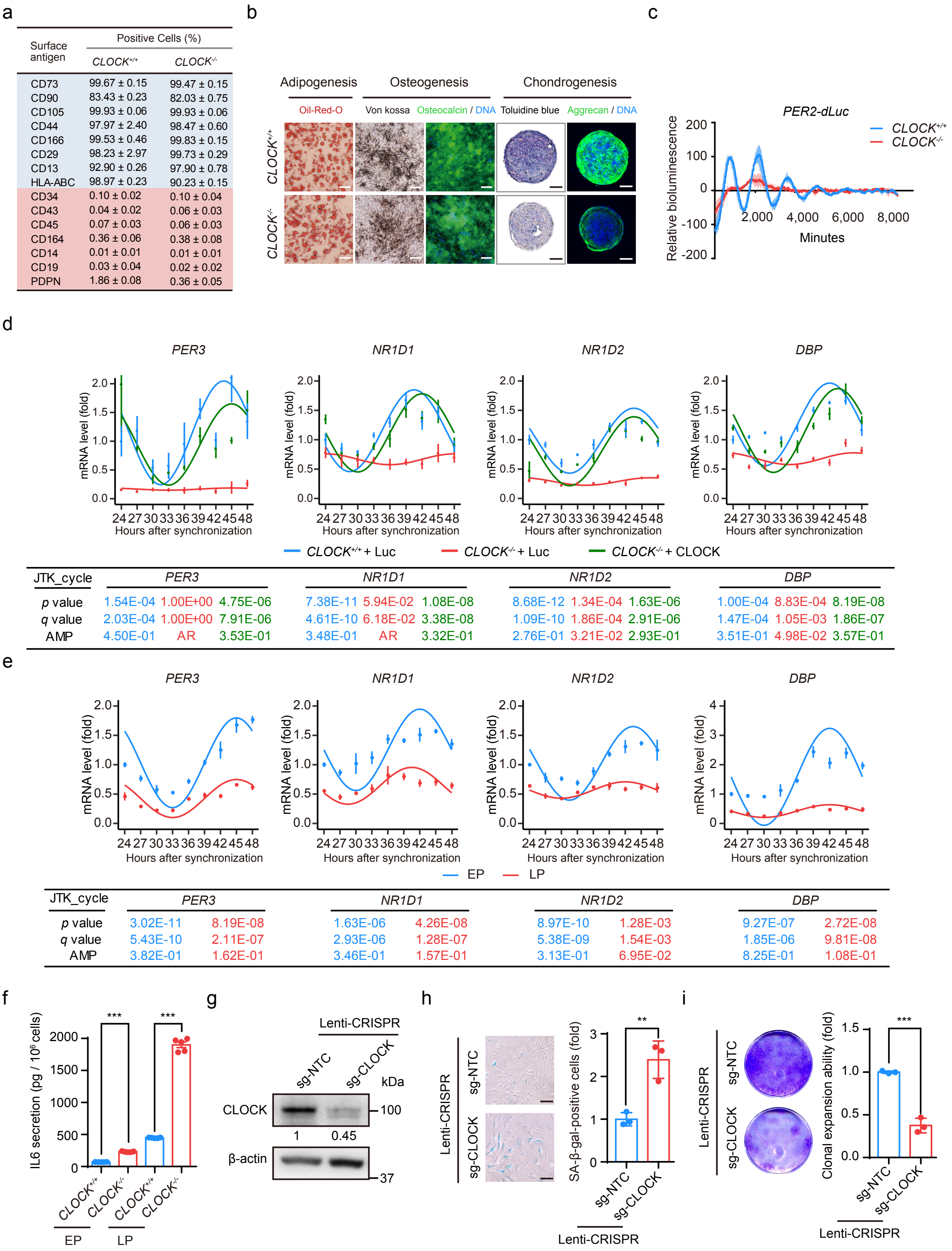

a

Surface antigen	Positive Cells (%) CLOCK+/+	CLOCK-/-
CD73	99.67 ± 0.15	99.47 ± 0.15
CD90	83.43 ± 0.23	82.03 ± 0.75
CD105	99.93 ± 0.06	99.93 ± 0.06
CD44	97.97 ± 2.40	98.47 ± 0.60
CD166	99.53 ± 0.46	99.83 ± 0.15
CD29	98.23 ± 2.97	99.73 ± 0.29
CD13	92.90 ± 0.26	97.90 ± 0.78
HLA-ABC	98.97 ± 0.23	90.23 ± 0.15
CD34	0.10 ± 0.02	0.10 ± 0.04
CD43	0.04 ± 0.02	0.06 ± 0.03
CD45	0.07 ± 0.03	0.06 ± 0.03
CD164	0.36 ± 0.06	0.38 ± 0.08
CD14	0.01 ± 0.01	0.01 ± 0.01
CD19	0.03 ± 0.04	0.02 ± 0.02
PDPN	1.86 ± 0.08	0.36 ± 0.05

d

JTK_cycle	PER3			NR1D1			NR1D2			DBP		
p value	1.54E-04	1.00E+00	4.75E-06	7.38E-11	5.94E-02	1.08E-08	8.68E-12	1.34E-04	1.63E-06	1.00E-04	8.83E-04	8.19E-08
q value	2.03E-04	1.00E+00	7.91E-06	4.61E-10	6.18E-02	3.38E-08	1.09E-10	1.86E-04	2.91E-06	1.47E-04	1.05E-03	1.86E-07
AMP	4.50E-01	AR	3.53E-01	3.48E-01	AR	3.32E-01	2.76E-01	3.21E-02	2.93E-01	3.51E-01	4.98E-02	3.57E-01

e

JTK_cycle	PER3		NR1D1		NR1D2		DBP	
p value	3.02E-11	8.19E-08	1.63E-06	4.26E-08	8.97E-10	1.28E-03	9.27E-07	2.72E-08
q value	5.43E-10	2.11E-07	2.93E-06	1.28E-07	5.38E-09	1.54E-03	1.85E-06	9.81E-08
AMP	3.82E-01	1.62E-01	3.46E-01	1.57E-01	3.13E-01	6.95E-02	8.25E-01	1.08E-01

Supplementary information, Fig. S2 Characterization of CLOCK-deficient hMSCs. **a** Surface antigen analysis of $CLOCK^{+/+}$ and $CLOCK^{-/-}$ hMSCs. Data are presented as means ± SEM. *n* = 3 biological replicates. **b** Characterization of the multilineage differentiation potential of $CLOCK^{+/+}$ and $CLOCK^{-/-}$ hMSCs. Toluidine blue and Aggrecan staining were used to evaluate chondrogenesis. Von Kossa and Osteocalcin staining were used to evaluate osteogenesis. Oil Red O staining were used to evaluate adipogenesis. Scale bars, 100 μm. **c** Representative traces of circadian oscillation in $CLOCK^{+/+}$ and $CLOCK^{-/-}$ hMSCs monitored by a transiently transfected reporter plasmid expressing destabilized Luc driven by the *PER2* gene promoter (*PER2-dLuc*). Continuous monitoring of Luc activity revealed significant amplitude dampening of PER2::LUC oscillations in $CLOCK^{-/-}$ hMSCs. Data are presented as means ± SD. *n* = 3 biological replicates. **d** Relative mRNA levels of the indicated genes from forskolin-synchronized $CLOCK^{+/+}$ and $CLOCK^{-/-}$ hMSCs transduced with lentiviruses expressing Luc or CLOCK measured at the indicated time points. The qPCR data shown above were further analyzed by JTK_Cycle analysis to determine the rhythmicity and amplitude of the peaks (Time course threads of $CLOCK^{+/+}$ or $CLOCK^{-/-}$ hMSCs with both *p* and *q* values < 0.05 were considered rhythmic). AR, arrhythmic. Data are presented as means ± SD. *n* = 4. The solid fitted line represents a cosinor-fitted curve that indicates rhythmic expression. Data are representative of two independent experiments. **e** Relative mRNA levels of the indicated genes from forskolin-synchronized EP (P4) and LP (P13) $CLOCK^{+/+}$ hMSCs measured at the indicated time points. The RT-qPCR data shown above were further analyzed by JTK_Cycle analysis to determine the rhythmicity and amplitude of the peaks (Time course threads of EP or LP hMSCs with both *p* and *q* values < 0.05 were considered rhythmic). AR, arrhythmic. Data are presented as means ± SD. *n* = 4. The solid fitted line represents a cosinor-fitted curve that indicates rhythmic expression. Data are representative of two independent experiments. **f** ELISA of IL6 secretion in EP (P4) and LP (P9) $CLOCK^{+/+}$ and $CLOCK^{-/-}$ hMSCs. Data are presented as means ± SEM. *n* = 5. ***p < 0.001 (Two-tailed unpaired Student's *t*-test). **g** Western blot analysis of CLOCK in $CLOCK^{+/+}$ hMSCs transduced with lentivirus-CRISPRv2 targeting CLOCK or an NTC sgRNA. β-actin was used as the loading control. Data are representative of three independent experiments. NTC means non-targeting control. **h** SA-β-gal staining in $CLOCK^{+/+}$ hMSCs transduced with lentivirus-CRISPRv2 targeting CLOCK or an NTC sgRNA. Data are presented as means ± SEM. *n* = 3 biological replicates. **p < 0.01 (Two-tailed unpaired Student's *t*-test). Scale bars, 100 μm. NTC means non-targeting control. **i** Clonal expansion assay of $CLOCK^{+/+}$ hMSCs transduced with lentivirus-CRISPRv2 targeting CLOCK or an NTC sgRNA. Data are presented as means ± SEM. *n* = 3 biological replicates. ***p < 0.001 (Two-tailed unpaired Student's *t*-test). NTC means non-targeting control.
