# Supplementary material for: Stabilization of heterochromatin by CLOCK promotes stem cell rejuvenation and cartilage regeneration
Source: Cell Res. 2020 Jul 31;31(2):187–205. doi: 10.1038/s41422-020-0385-7 (PMC8027439; doi:10.1038/s41422-020-0385-7)
Supplement: Supplementary file 3 — Figure S3 [file 41422_2020_385_MOESM3_ESM.pdf]

Figure S3

a

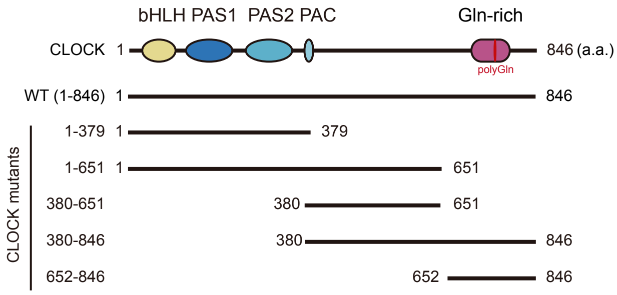

b

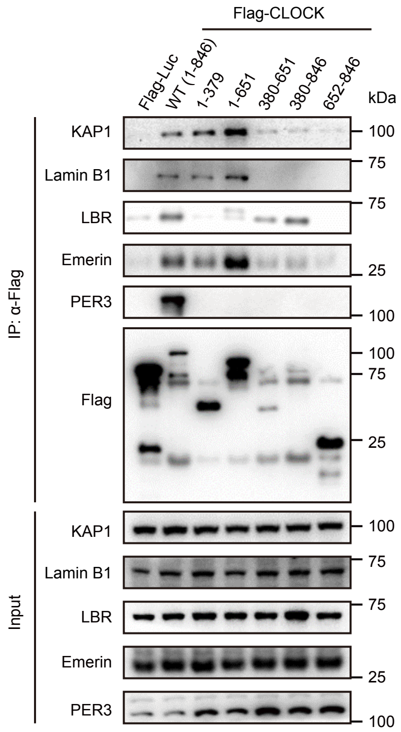

c

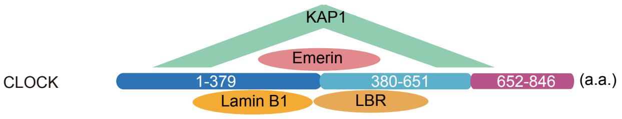

d

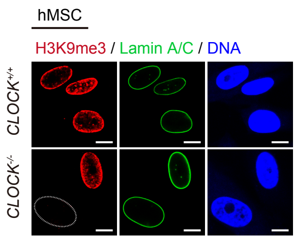

Supplementary information, Fig. S3 Mapping of CLOCK domains interacting with nuclear lamina proteins and KAP1. a Schematic diagram of different CLOCK truncations. **b** Co-IP of KAP1 and nuclear lamina proteins with exogenous full-length and truncated CLOCK proteins in HEK293T cells. Data are representative of two independent experiments. **c** Schematic diagram of a truncated CLOCK protein (a.a. 1-846) and the possible domains interacting with KAP1, LBR and Emerin according to the co-IP results shown in Supplementary information, Fig. S3b. **d** Representative images of H3K9me3 staining of late-passage $CLOCK^{+/+}$ and $CLOCK^{-/-}$ hMSCs (P9). Dashed lines indicate the nuclear boundaries of cells with decreased signal of H3K9me3. Related to Fig. 2f, g. Scale bars, 10 μm.
