# Supplementary material for: Stabilization of heterochromatin by CLOCK promotes stem cell rejuvenation and cartilage regeneration
Source: Cell Res. 2020 Jul 31;31(2):187–205. doi: 10.1038/s41422-020-0385-7 (PMC8027439; doi:10.1038/s41422-020-0385-7)
Supplement: Supplementary file 4 — Figure S4 [file 41422_2020_385_MOESM4_ESM.pdf]

Figure S4

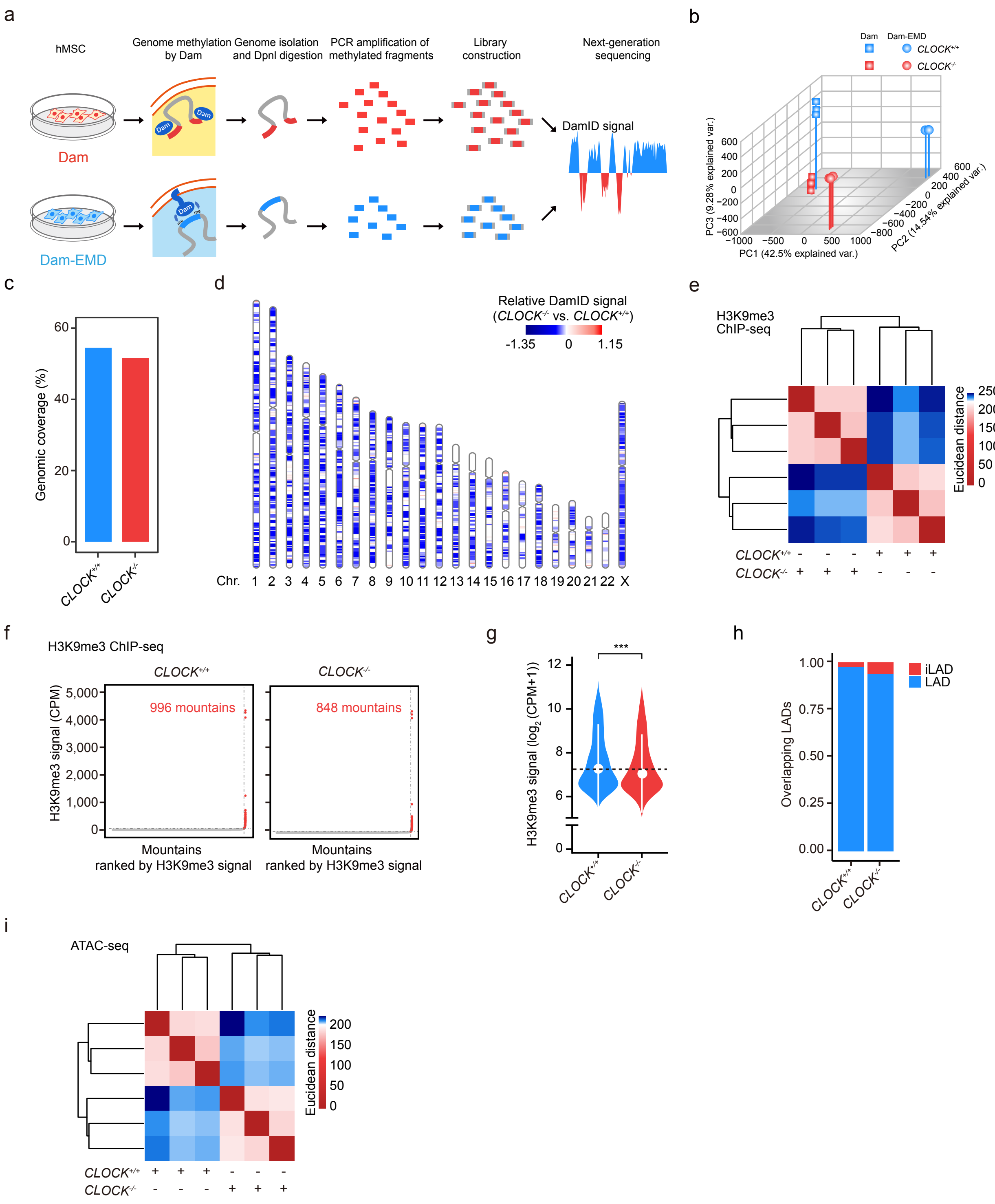
a
hMSC
Genome methylation by Dam
Genome isolation and DpnI digestion
PCR amplification of methylated fragments
Library construction
Next-generation sequencing
Dam
Dam-EMD
DamID signal
b
Dam
Dam-EMD
CLOCK+/+
CLOCK-/-
PC1 (42.5% explained var.)
PC2 (14.54% explained var.)
PC3 (9.28% explained var.)
c
Genomic coverage (%)
CLOCK+/+
CLOCK-/-
d
Relative DamID signal
(CLOCK-/- vs. CLOCK+/+)
-1.35
0
1.15
Chr. 1 2 3 4 5 6 7 8 9 10 11 12 13 14 15 16 17 18 19 20 21 22 X
e
H3K9me3 ChIP-seq
Eucidean distance
CLOCK+/+ - - - + + +
CLOCK-/- + + + - - -
f
H3K9me3 ChIP-seq
CLOCK+/+
CLOCK-/-
996 mountains
848 mountains
H3K9me3 signal (CPM)
Mountains ranked by H3K9me3 signal
g
H3K9me3 signal (log2 (CPM+1))
***
h
Overlapping LADs
iLAD
LAD
i
ATAC-seq
Eucidean distance
CLOCK+/+ + + + - - -
CLOCK-/- - - - + + +

Supplementary information, Fig. S4 Epigenomic analyses of *CLOCK*$^{-/-}$ hMSCs. a The flow chart of the DamID-seq strategy. **b** Principal component analysis to assess the reproducibility of DamID-seq data in late-passage *CLOCK*$^{+/+}$ and *CLOCK*$^{-/-}$ hMSCs (P9). **c** Bar chart showing the genomic coverage of LADs in late-passage *CLOCK*$^{+/+}$ and *CLOCK*$^{-/-}$ hMSCs (P9). **d** Chromosome ideogram showing the relative DamID signals at LAD regions of *CLOCK*$^{-/-}$ hMSCs compared to those of late-passage *CLOCK*$^{+/+}$ hMSCs (P9) across the 23 pairs of chromosomes. The color key from blue to red indicates low to high of the relative DamID signals at each LAD region, respectively. **e** Heatmap showing the Euclidean distance analysis to assess the reproducibility of H3K9me3 ChIP-seq data in late-passage *CLOCK*$^{+/+}$ and *CLOCK*$^{-/-}$ hMSCs (P9). The color key of the Euclidean distance from red to blue indicates strong to weak correlation, respectively. **f** Distribution of the H3K9me3 ChIP-seq signal at H3K9me3 peaks revealed two classes of H3K9me3 peaks in late-passage *CLOCK*$^{+/+}$ and *CLOCK*$^{-/-}$ hMSCs (P9). H3K9me3 peaks are plotted in increasing order based on the H3K9me3 signal. H3K9me3 mountains are defined as H3K9me3 peaks above the inflection points of the curves. **g** Violin plot showing the H3K9me3 signals at H3K9me3 mountains in late-passage *CLOCK*$^{+/+}$ and *CLOCK*$^{-/-}$ hMSCs (P9). The white circles represent the median values, and the white lines represent the values within the IQR from smallest to largest. ***$p < 0.001$ (Two-sided Wilcoxon rank-sum test). **h** Bar plot showing the percentages of H3K9me3 mountains located in LAD regions in late-passage *CLOCK*$^{+/+}$ and *CLOCK*$^{-/-}$ hMSCs (P9). **i** Heatmap showing the Euclidean distance analysis to determine the reproducibility of ATAC-seq data in late-passage *CLOCK*$^{+/+}$ and *CLOCK*$^{-/-}$ hMSCs (P9). The color key of the Euclidean distance from red to blue indicates strong to weak correlation, respectively.
