# Supplementary material for: Stabilization of heterochromatin by CLOCK promotes stem cell rejuvenation and cartilage regeneration
Source: Cell Res. 2020 Jul 31;31(2):187–205. doi: 10.1038/s41422-020-0385-7 (PMC8027439; doi:10.1038/s41422-020-0385-7)
Supplement: Supplementary file 5 — Figure S5 [file 41422_2020_385_MOESM5_ESM.pdf]

Figure S5

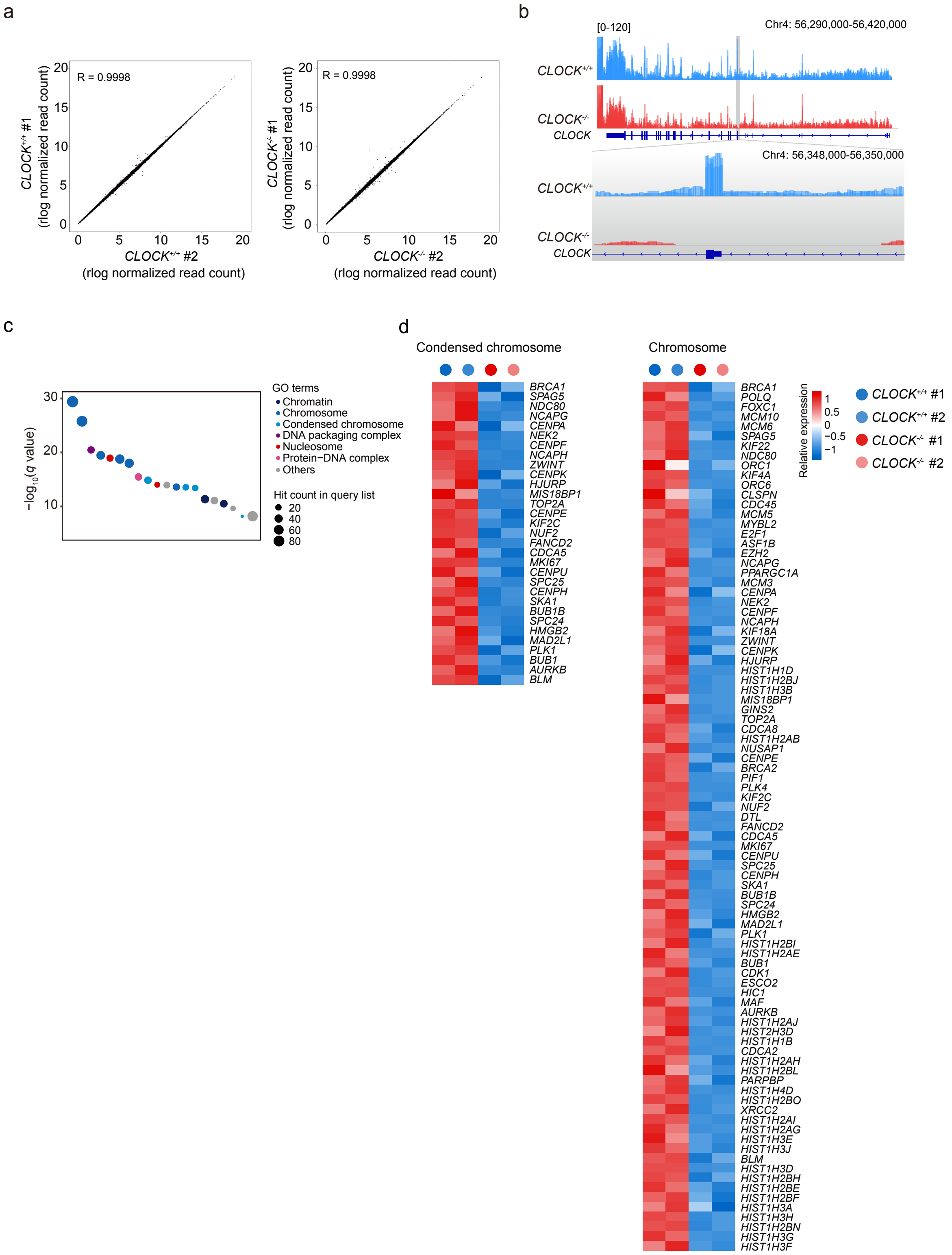

a
R = 0.9998
CLOCK+/+ #1 (rlog normalized read count)
CLOCK+/+ #2 (rlog normalized read count)
R = 0.9998
CLOCK-/- #1 (rlog normalized read count)
CLOCK-/- #2 (rlog normalized read count)
b
[0-120]
Chr4: 56,290,000-56,420,000
CLOCK+/+
CLOCK-/-
CLOCK
Chr4: 56,348,000-56,350,000
CLOCK+/+
CLOCK-/-
CLOCK
c
−log10(q value)
GO terms
Chromatin
Chromosome
Condensed chromosome
DNA packaging complex
Nucleosome
Protein−DNA complex
Others
Hit count in query list
20
40
60
80
d
Condensed chromosome
BRCA1 SPAG5 NDC80 NCAPG CENPA NEK2 CENPF NCAPH ZWINT CENPK HJURP MIS18BP1 TOP2A CENPE KIF2C NUF2 FANCD2 CDCA5 MKI67 CENPU SPC25 CENPH SKA1 BUB1B SPC24 HMGB2 MAD2L1 PLK1 BUB1 AURKB BLM
Chromosome
BRCA1 POLQ FOXC1 MCM10 MCM6 SPAG5 KIF22 NDC80 ORC1 KIF4A ORC6 CLSPN CDC45 MCM5 MYBL2 E2F1 ASF1B EZH2 NCAPG PPARGC1A MCM3 CENPA NEK2 CENPF NCAPH KIF18A ZWINT CENPK HJURP HIST1H1D HIST1H2BJ HIST1H3B MIS18BP1 GINS2 TOP2A CDCA8 HIST1H2AB NUSAP1 CENPE BRCA2 PIF1 PLK4 KIF2C NUF2 DTL FANCD2 CDCA5 MKI67 CENPU SPC25 CENPH SKA1 BUB1B SPC24 HMGB2 MAD2L1 PLK1 HIST1H2BI HIST1H2AE BUB1 CDK1 ESCO2 HIC1 MAF AURKB HIST1H2AJ HIST2H3D HIST1H1B CDCA2 HIST1H2AH HIST1H2BL PARPBP HIST1H4D HIST1H2BO XRCC2 HIST1H2AI HIST1H2AG HIST1H3E HIST1H3J BLM HIST1H3D HIST1H2BH HIST1H2BE HIST1H2BF HIST1H3A HIST1H3H HIST1H2BN HIST1H3G HIST1H3F
Relative expression
1
0.5
0
−0.5
−1
CLOCK+/+ #1
CLOCK+/+ #2
CLOCK-/- #1
CLOCK-/- #2

Supplementary information, Fig. S5 RNA-seq analysis of *CLOCK*$^{-/-}$ hMSCs. a Scatter plots showing the correlation between RNA-seq replicates from late-passage *CLOCK*$^{+/+}$ and *CLOCK*$^{-/-}$ hMSCs (P9). **b** Top, representative tracks showing the RNA level of *CLOCK* in late-passage *CLOCK*$^{+/+}$ and *CLOCK*$^{-/-}$ hMSCs (P9). Bottom, gene editing sites for *CLOCK* (exon 5) using CRISPR/Cas9-mediated HR with its flanking region zoomed. **c** Scatter plot showing the GO biological process terms enriched in downregulated genes in *CLOCK*$^{-/-}$ hMSCs compared to *CLOCK*$^{+/+}$ hMSCs at late-passage (P9). **d** Heatmap showing the Z-score normalized expression levels of genes associated with “Condensed chromatin” and “Chromosome” in *CLOCK*$^{-/-}$ hMSCs compared to *CLOCK*$^{+/+}$ hMSCs at late-passage (P9).
