# Supplementary material for: Stabilization of heterochromatin by CLOCK promotes stem cell rejuvenation and cartilage regeneration
Source: Cell Res. 2020 Jul 31;31(2):187–205. doi: 10.1038/s41422-020-0385-7 (PMC8027439; doi:10.1038/s41422-020-0385-7)
Supplement: Supplementary file 6 — Figure S6 [file 41422_2020_385_MOESM6_ESM.pdf]

Figure S6

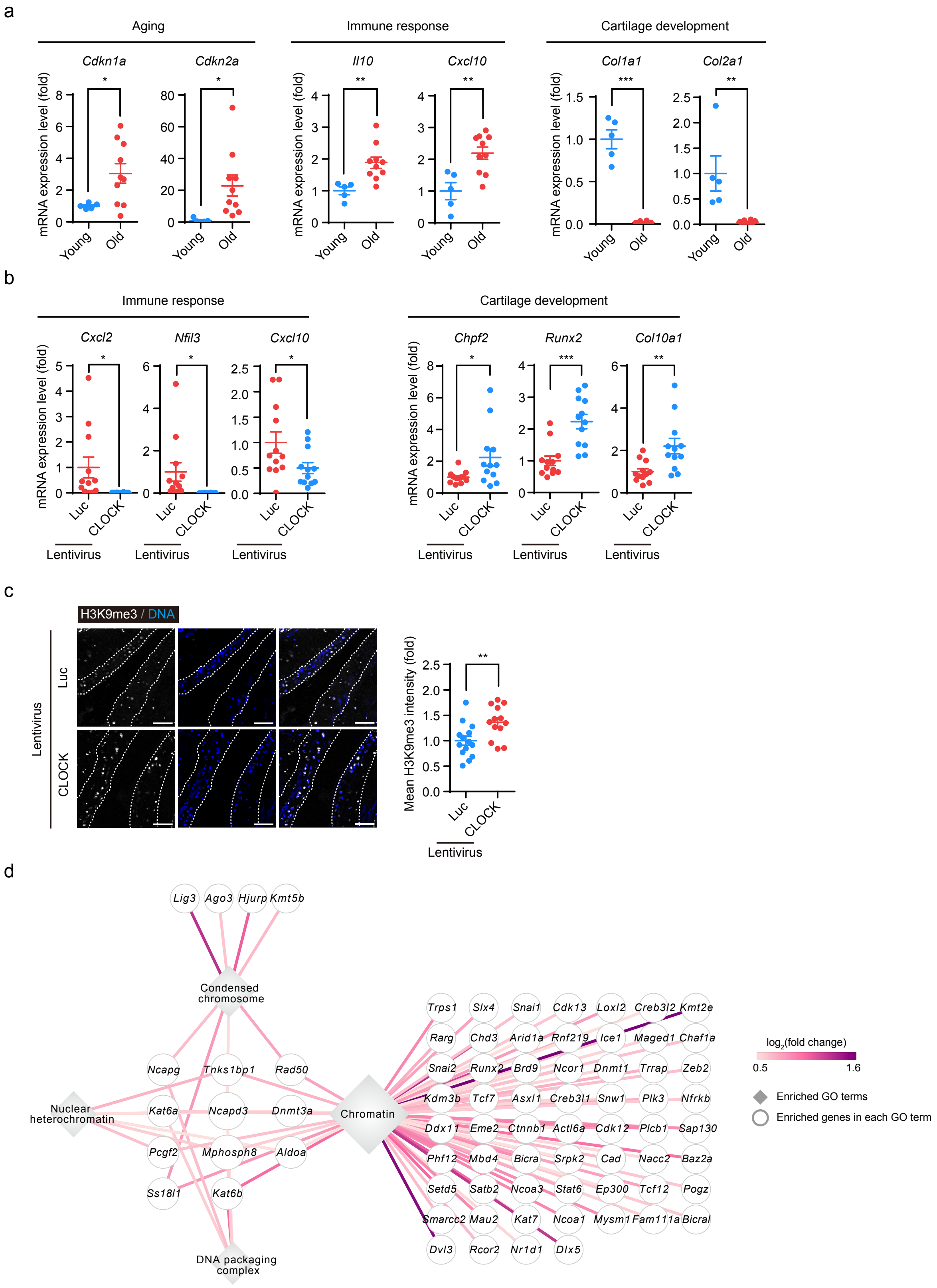
a
Aging
Immune response
Cartilage development
Cdkn1a
Cdkn2a
Il10
Cxcl10
Col1a1
Col2a1
mRNA expression level (fold)
Young
Old
b
Immune response
Cartilage development
Cxcl2
Nfil3
Cxcl10
Chpf2
Runx2
Col10a1
mRNA expression level (fold)
Luc
CLOCK
Lentivirus
c
H3K9me3 / DNA
Luc
CLOCK
Lentivirus
Mean H3K9me3 intensity (fold)
d
Condensed chromosome
Nuclear heterochromatin
DNA packaging complex
Chromatin
Lig3
Ago3
Hjurp
Kmt5b
Ncapg
Tnks1bp1
Rad50
Kat6a
Ncapd3
Dnmt3a
Pcgf2
Mphosph8
Aldoa
Ss18l1
Kat6b
Trps1
Slx4
Snai1
Cdk13
Loxl2
Creb3l2
Kmt2e
Rarg
Chd3
Arid1a
Rnf219
Ice1
Maged1
Chaf1a
Snai2
Runx2
Brd9
Ncor1
Dnmt1
Trrap
Zeb2
Kdm3b
Tcf7
Asxl1
Creb3l1
Snw1
Plk3
Nfrkb
Ddx11
Eme2
Ctnnb1
Actl6a
Cdk12
Plcb1
Sap130
Phf12
Mbd4
Bicra
Srpk2
Cad
Nacc2
Baz2a
Setd5
Satb2
Ncoa3
Stat6
Ep300
Tcf12
Pogz
Smarcc2
Mau2
Kat7
Ncoa1
Mysm1
Fam111a
Bicral
Dvl3
Rcor2
Nr1d1
Dlx5
log2(fold change)
Enriched GO terms
Enriched genes in each GO term

Supplementary information, Fig. S6 CLOCK alleviates osteoarthritis of aged mice. a RT-qPCR analysis of the indicated genes in the joints of young mice (n = 5 mice) and old mice (n = 10 mice). Data are presented as means ± SEM. *p < 0.05; **p < 0.01; ***p < 0.001 (Two-tailed unpaired Student's t-test). **b** RT-qPCR analysis results of the indicated genes in the joints of aged mice injected with lentiviruses expressing Luc or CLOCK. Luc (n = 12 mice), CLOCK (n = 12 mice). Data are presented as means ± SEM. *p < 0.05; **p < 0.01; ***p < 0.001 (Two-tailed unpaired Student's t-test). **c** H3K9me3 staining of articular cartilage from aged mice injected with lentiviruses expressing Luc (n = 14 mice) or CLOCK (n = 13 mice). Quantitative data to the right are presented as means ± SEM. Dashed lines indicates the articular cartilage surface in the joints of aged mice injected with lentiviruses expressing Luc or CLOCK. **p < 0.01 (Two-tailed unpaired Student's t-test). Scale bars, 50 μm. **d** Network showing the expression levels for upregulated genes involved in "Condensed chromosome", "Nuclear heterochromatin", "DNA packaging complex" and "Chromatin" in the joints of aged mice injected with lentiviruses expressing CLOCK.
